# Metabolic rewiring driven by phosphoglycolate phosphatase deletion inhibits ferroptosis

**DOI:** 10.1126/sciadv.aeb2368

**Published:** 2026-05-29

**Authors:** Marian Brenner, Sina Höhlein, Paul Wirth, Leon Neidt, Melina Lappe, Elisa Hopke, Eirini Sfakianaki, Martina Fischer, Kerstin Hadamek, Angelika Keller, Sebastian Bothe, José Pedro Friedmann Angeli, Anna M. Schmoker, Arminja N. Kettenbach, Agnes Fekete, Werner Schmitz, Ingrid Tessmer, Elisabeth Jeanclos, Antje Gohla

**Affiliations:** ^1^ Institute for Pharmacology and Toxicology, University of Würzburg, Germany.; ^2^ Rudolf-Virchow-Zentrum—Center for Integrative and Translational Bioimaging, University of Würzburg, Germany.; ^3^ Institute of Pharmacy and Food Chemistry, University of Würzburg, Germany.; ^4^ Dartmouth Cancer Center, Lebanon, NH, USA.; ^5^ Department of Biochemistry and Cell Biology, Geisel School of Medicine at Dartmouth, Hanover, NH, USA.; ^6^ Pharmaceutical Biology, Julius von Sachs Institute and Biocenter, University of Würzburg, Germany.; ^7^ Department of Biochemistry and Molecular Biology, Theodor Boveri Institute, Biocenter, University of Würzburg, Germany.

## Abstract

Modulating ferroptosis, a form of cell death driven by uncontrolled lipid peroxidation, is of interest in numerous diseases. Here, we found that the deletion of phosphoglycolate phosphatase (PGP), an essential enzyme that safeguards high glycolytic flux, suppresses ferroptosis. Using metabolomic and isotopic labeling experiments together with lipid and proteomic profiling, we find that PGP loss drives a rewiring of the pentose phosphate pathway and of cellular energy and lipid metabolism that triggers a multifactorial antioxidant response. Paradoxically, our attempts to block PGP pharmacologically led to the realization that the recently described PGP inhibitor compound 1 (CP1) exerts a strong ferroptosis-sensitizing effect. Using genetic, biochemical, and biophysical approaches, we characterize CP1 as a direct, species-independent, dual inhibitor of PGP and ferroptosis suppressor protein 1 (FSP1), and further find that CP1 triggers FSP1 self-assembly. In sum, we identify PGP as a target protein for ferroptosis control and introduce a small-molecule FSP1 inhibitor with unique features to the armamentarium of pharmacological ferroptosis modulators.

## Teaser

Phosphoglycolate phosphatase loss rewires cellular metabolism and triggers an antioxidant response that mitigates ferroptosis.

## INTRODUCTION

**Fig. 1.**
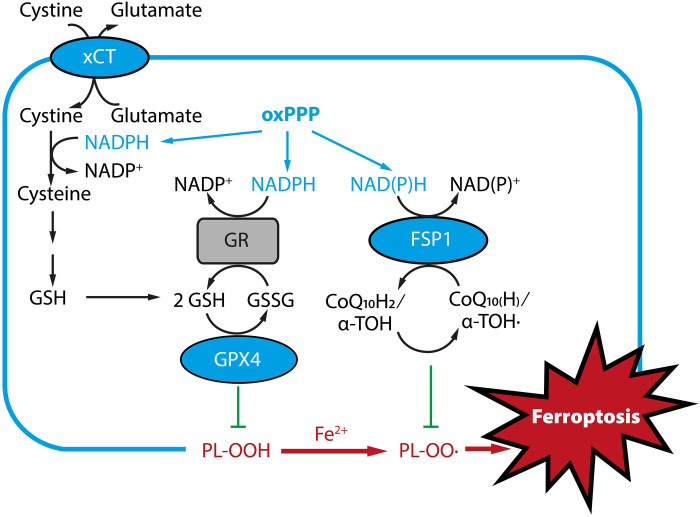
Ferroptotic cell death and key ferroptosis suppressors. xCT, cystine/glutamate antiporter; GPX4, glutathione peroxidase-4, FSP1, ferroptosis suppressor protein; GSH/GSSG, glutathione/glutathione disulfide; GR, glutathione reductase; oxPPP, oxidative part of the pentose phosphate pathway; CoQ_10_H_2_/CoQ_10_(H), reduced/oxidized coenzyme Q10 (ubiquinol/ubichinone); α-TOH/α-TOH·, α-tocopherol/α-tocopheryl radical; PL─OOH/PL─OO·, phospholipid hydroperoxide/phospholipid peroxyl radical.

Triggering oxidative cell death is an important component of multiple cytotoxic drugs, including many used in oncology practice (*1*–*4*). Recently, ferroptosis has attracted interest as a nonapoptotic form of oxidative cell death (*5*, *6*). Ferroptosis is driven by the iron-dependent buildup of destructive lipid peroxides, lastly leading to membrane rupture and cell death (Fig. 1). Numerous factors contribute to the induction and execution of ferroptosis, including cellular metabolism, redox homeostasis, nutrients, and inputs from extra- and intracellular signaling and environmental stressors (*7*). This form of cell death is therefore under constant surveillance by several cell-intrinsic ferroptosis-suppressing pathways, which might offer novel pharmacological entry points into treatment-resistant cancers (*8*–*12*). Conversely, the inhibition of ferroptosis holds promise for the therapy of neurodegenerative diseases (*13*) or ischemia/reperfusion injuries (*7*)

A key antiferroptotic axis involves cystine uptake via the cystine/glutamate antiporter (xCT, also known as system x_c_^−^) and cysteine-dependent glutathione (GSH) biosynthesis (*14*). GSH, the most abundant reductant in mammalian cells, is a cofactor of the major cellular ferroptosis suppressor glutathione peroxidase 4 (GPX4), a selenoprotein that reduces phospholipid hydroperoxides to their corresponding phospholipid alcohols (*15*). In addition to GPX4, the ferroptosis suppressor protein 1 (FSP1, gene name: *AIFM2*) counteracts deleterious lipid peroxidation by regenerating the ubiquinol and α-tocopherol antioxidant systems (Fig. 1) (*16*, *17*). Important ferroptosis surveillance mechanisms continue to be found, including the di/tetrahydrobiopterin or squalene-mediated inhibition of lipid peroxidation (*18*, *19*), DHODH-mediated lipid peroxide detoxification in mitochondria (*20*), the antiferroptotic cholesterol synthesis pathway (*21*, *22*), or the peroxiredoxin-6 selenide shuttle that supports the synthesis of selenoproteins such as GPX4 (*23*, *24*).

**Fig. 2.**
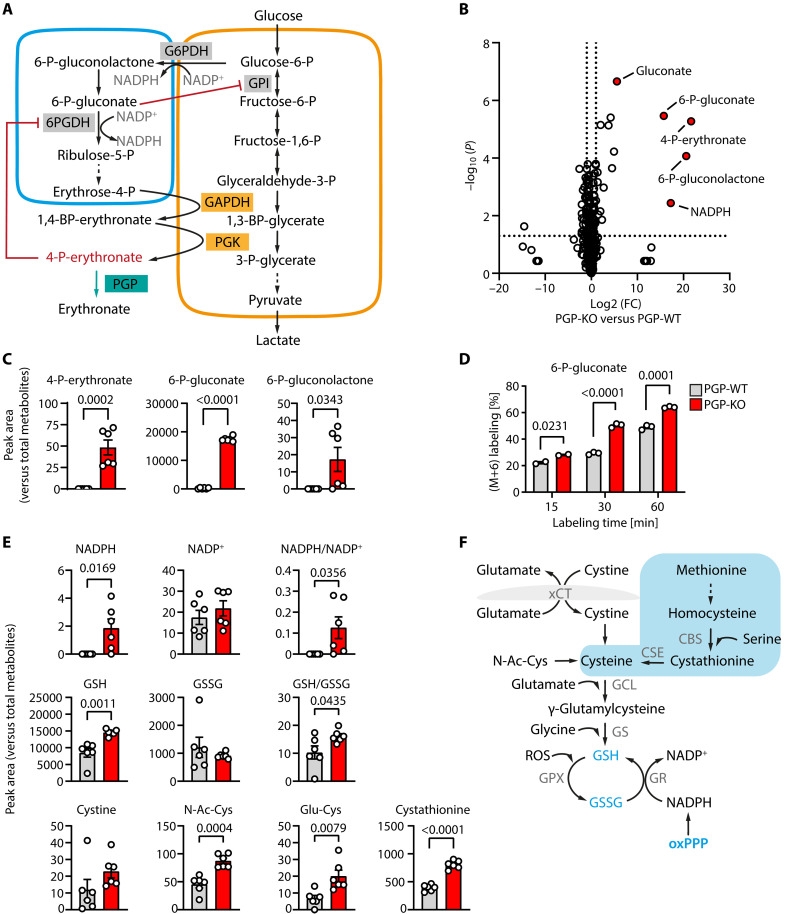
Impact of PGP loss on glucose flux into the PPP and on the GSH antioxidant system. (**A**) Role of PGP in maintaining flux through the PPP pathway (blue box). The enzyme glyceraldehyde-3-P dehydrogenase (GAPDH) in glycolysis (yellow box) can erroneously act on erythrose-4-P, a metabolite of the PPP and a structural analog of the GAPDH substrate glyceraldehyde-3P. The resulting intermediate, 1,4-bisphosphoerythronate (1,4-BP-erythronate), is converted to 4-P-erythronate (4PE) via the activities of acylphosphatase 1 (not shown) and/or phosphoglycerate kinase (PGK). PGP loss leads to an accumulation of 4PE, a potent inhibitor of 6-phosphogluconate dehydrogenase (6PGDH). The accumulation of 6-phosphogluconate (6PG) can result in the inhibition of glucose-6-P-isomerase (GPI). (**B**) Volcano plot analysis of a representative untargeted metabolomic comparison of *PGP*-WT and *PGP*-KO HT1080 cells. (**C**) Relative abundance of the indicated metabolites, analyzed by mass spectrometry. Data are mean values ± SE of *n* = 6 biologically independent experiments. (**D**) Isotopic tracing experiments. *PGP*-WT or *PGP*-KO HT1080 cells were incubated with ^13^C_6_-labeled glucose for the indicated times and analyzed by mass spectrometry. Data are means ± SE of *n* = 2 to 3 biologically independent experiments. (**E**) Relative abundance of the indicated metabolites. Data are means ± SE of *n* = 6 biologically independent experiments. N-Ac-Cys, *N*-acetylcysteine; Glu-Cys, γ-glutamylcysteine. Statistical analysis in (C), (D), and (E): Unpaired, two-sided *t* tests; *P* values are indicated. (**F**) Schematic overview of GSH biosynthesis and regeneration. The transsulfuration pathway is shaded in light blue. CBS, cystathionine β-synthase; CSE, cystathionine γ-lyase; GCL, glutamate-cysteine ligase; GS, glutathione synthase.

In most cancer cells, the GSH antioxidant system is maintained by GSH reductase and NADPH [reduced form of nicotinamide adenine dinucleotide phosphate (NADP^+^)] derived from the oxidative part of the pentose phosphate pathway (oxPPP) (*25*, *26*) and is coupled to xCT-mediated cystine uptake (*27*). It was noted from the outset of ferroptosis research that inhibiting or silencing the core oxPPP enzyme 6-phosphogluconate dehydrogenase (6PGDH; Fig. 2A) protects against ferroptosis (*14*). Consistent with this observation, 6PGDH was later identified as a ferroptosis driver in lung adenocarcinoma (*28*). Genome-wide short hairpin RNA (shRNA) and CRISPR screens for modifiers of oxidative stress sensitivity in cancer cells independently showed that perturbing the oxPPP by deleting 6PGDH results in a strong protection against oxidative stress (*29*). These CRISPR knockout (KO) screens also identified phosphoglycolate phosphatase (PGP) as a protective factor, but its role was not further investigated.

PGP (also known as glycerol 3-phosphate phosphatase or AUM) (*30*, *31*) is a haloacid dehalogenase-type phosphatase (*32*) that safeguards glycolytic and PPP flux (*33*, *34*), and *PGP*-KO can result in 6PGDH inhibition (Fig. 2A) (*34*). PGP deletion leads to a shutdown of glycolysis and a metabolic gridlock in central carbon metabolism, suggesting that pharmacological PGP inhibitors may be beneficial in combination therapies of highly glycolytic tumors (*35*). Recent work has demonstrated that both PGP overexpression and PGP deletion can reduce experimental tumor growth in vivo (*36*). Nevertheless, how PGP targeting confers oxidative stress protection remains unexplored (*29*).

The aim of the present manuscript was to address this knowledge gap. Using untargeted metabolomics, isotopic labeling experiments, proteomics, and cellular lipid analyses together with functional cellular assays, we find that PGP deletion can suppress ferroptosis, without generally preventing acute oxidative cell death. Mechanistically, we show that the loss of PGP activity necessitates a metabolic rewiring to prevent cellular energy stress and that this metabolic compensation triggers a multifactorial antioxidative stress response, possibly involving the activation of nuclear factor erythroid 2-related factor 2 (NFE2L2/NRF2) together with changes in membrane lipids. Our attempts to inhibit PGP pharmacologically paradoxically showed that the recently described PGP inhibitor compound 1 (CP1) exerts a strong ferroptosis-sensitizing effect. We identify CP1 as a dual, direct inhibitor of both murine and human PGP and FSP1 and additionally find that CP1 triggers FSP1 self-assembly. These properties of CP1 may be useful in the future development of molecules that inhibit FSP1 by sequestering it from membranes.

## RESULTS

### PGP deletion fuels the GSH antioxidant system

The oxPPP plays a pivotal role in oxidative stress protection (*26*). PGP loss has been shown to cause a block in the oxPPP due to a buildup of the PGP substrate 4-phosphoerythronate [4-P-erythronate (4PE)], derived from off-target glycolytic metabolism (Fig. 2A) (*34*). However, genome-wide CRISPR KO screens for oxidative stress modifiers suggested PGP as a protective factor (*29*). To explore the mechanism underlying this seemingly paradoxical effect, we first conducted mass spectrometry–based, untargeted metabolomic analyses in PGP wildtype (*PGP*-WT) and *PGP*-KO HT1080 human fibrosarcoma cells (fig. S1A).

The analysis of differentially abundant metabolites revealed that the PGP substrate 4PE together with oxPPP metabolites and NADPH were among the most highly up-regulated metabolites in PGP-deficient cells (Fig. 2B). 4PE is a potent inhibitor of 6PGDH with a *K*_i_ < 1 μM (*37*). Consistent with a 4PE-mediated oxPPP block at the level of 6PGDH (Fig. 2A), the loss of PGP led to a marked accumulation of the 6PGDH substrate 6-phosphogluconate (6PG) and its precursor 6-phosphogluconolactone (Fig. 2, B and C). While an increase in 6PG levels could in principle also arise from direct, PGP-mediated 6PG dephosphorylation, both the very high *K*_M_ of PGP for 6PG (2.67 mM; fig. S1B) and the fact that gluconate levels were increased in the absence of PGP (Fig. 2B and table S1) argue against this possibility. Consistent with previous findings, we also observed an increase in the PGP substrate 2-phospho-l-lactate (2PL; table S1), which can impair glycolytic flux by inhibiting phosphofructokinase-2 (*34*). Furthermore, we detected lower pyruvate and lactate levels in *PGP*-KO cells (table S1). In contrast, the levels of the PGP substrate glycerol-3-phosphate (*38*) were not increased in this model system (table S1). Thus, in line with previous observations in other cell types (*34*), PGP deletion in HT1080 cells blocks glycolysis and the oxPPP. Similar metabolic changes were recapitulated using a small interfering RNA (siRNA)–mediated *PGP* knockdown (fig. S1C and table S1).

OxPPP inhibition at the level of 6PGDH results in the buildup of 6PG, a classical inhibitor of glucose 6-phosphate isomerase (GPI) in upper glycolysis (Fig. 2A) (*39*). 6PG can thereby shift the equilibrium between fructose 6-phosphate/glucose 6-phosphate isomerization toward glucose 6-phosphate and reroute glucose flux toward oxPPP (*29*, *39*). To test this directly in our system, we conducted isotopic tracing experiments with ^13^C_6_-labeled glucose. Figure 2D shows that the time-dependent incorporation of ^13^C-labeled carbons into 6PG (M + 6) was significantly higher in *PGP*-KO than in *PGP*-WT cells. 6-Phosphogluconolactone could not be evaluated because it was below the detection limit in *PGP*-WT cells. Together with the substantial increase of 6PG in *PGP*-KO cells (Fig. 2C), these data strongly suggest that the flux from glucose to 6PG is much higher in the absence of PGP. Thus, the loss of PGP activity indeed causes a block in the oxPPP at the level of 6PGDH (*34*), but this block triggers a metabolic rewiring in upper glycolysis and reroutes glucose catabolism into the oxPPP. Nevertheless, we note that these data do not directly demonstrate increased glucose 6-phosphate dehydrogenase (G6PDH) flux.

To investigate the functional consequences of this metabolic rewiring, we considered that the oxPPP is a major source of cytosolic NADPH and that a rerouting of glucose 6-phosphate to feed the oxPPP can occur in cells with a very high NADPH demand (*26*). PGP deletion seems to increase NADPH levels (Fig. 2, B and E) and the NADPH/NADP^+^ ratio (Fig. 2E). These results should nevertheless be regarded as tentative, because NADPH levels detected with our mass spectrometry protocol were very low in *PGP*-KO cells and below the detection limit in *PGP*-WT cells (table S1). Still, cytosolic NADPH is known to be essential for GSH regeneration, and metabolites involved in GSH biosynthesis (Fig. 2F) were robustly detectable in our system. We found that GSH levels and the GSH/oxidized glutathione (GSSG) ratio were increased in *PGP*-KO cells (Fig. 2E). In addition, levels of the direct GSH precursors cystine, *N*-acetylcysteine, and γ-glutamylcysteine were higher in *PGP*-KO cells (Fig. 2E), whereas most amino acids, and all other acetylated or glutamylated amino acids were unaltered (table S1). In addition to transporter-mediated cystine uptake, cellular cysteine can also be synthesized from methionine as a sulfur donor through the transsulfuration pathway (Fig. 2F) (*40*). The analysis of metabolites in this pathway revealed elevated levels of the cysteine precursor cystathionine in *PGP*-KO cells (Fig. 2E and table S1). Together, these results demonstrate that PGP deficiency leads to a rerouting of glucose flux into the oxPPP. Our data also suggest that this metabolic rewiring could contribute to elevated GSH regeneration capacity and GSH biosynthesis.

### PGP deletion selectively suppresses ferroptosis

**Fig. 3.**
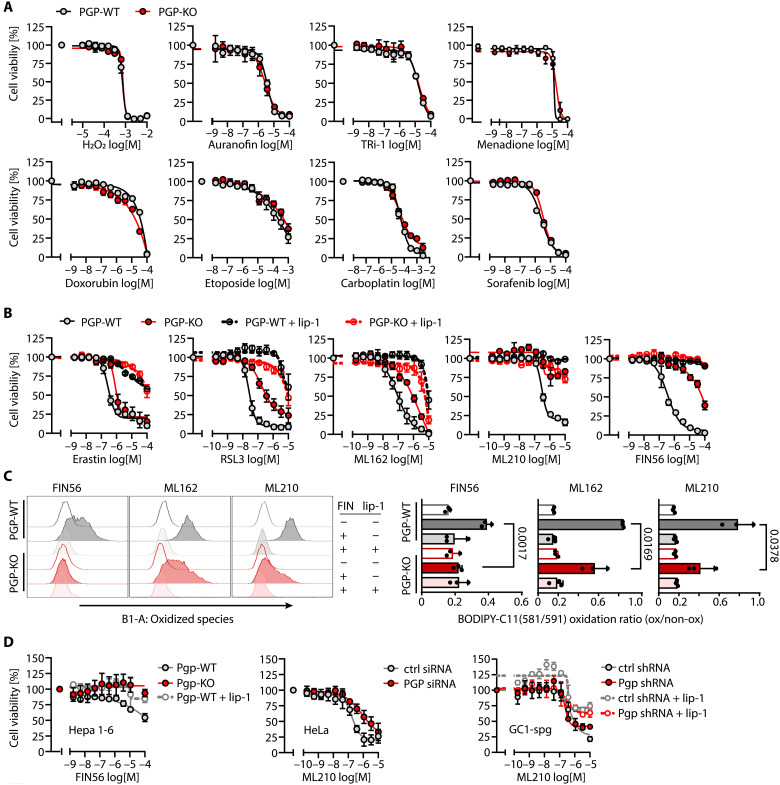
Effects of PGP deletion on oxidative or ferroptotic cell death. *PGP*-WT or *PGP*-KO HT1080 cells were incubated for 16 hours with serial dilutions of (**A**) hydrogen peroxide (H_2_O_2_) or the indicated, ROS-producing chemotherapeutics, or (**B**) with serial dilutions of the indicated ferroptosis inducers in the absence or presence of the ferroptosis inhibitor liproxstatin-1 (lip-1; 100 nM). (**C**) Effect of PGP loss on cellular lipid peroxidation investigated by flow cytometry using C11-BODIPY (581/591). HT1080 *PGP*-WT or *PGP*-KO cells were treated with FIN56 (5 μM), ML162 (0.25 μM), or ML210 (0.3 μM) in the absence [0.1% (v/v) DMSO] or presence of lip-1 (100 nM). Left: Representative histograms. Right: Summary data. Statistical testing: Unpaired, two-sided *t* tests; *P* values are indicated. (**D**) *Pgp*-WT or *Pgp*-KO Hepa 1-6 cells or *PGP*/*Pgp*-depleted HeLa or GC1-spg cells were incubated for 16 hours with serial dilutions of the indicated GPX4 inhibitors in the absence or presence of lip-1 (100 nM). [(A) to (D)] Cell viability was analyzed using resazurin. Except for the histograms in (C), all data are mean values ± SE of *n* = 3 biologically independent experiments. Apparently missing error bars are hidden by the symbols. ctrl, control.

The initial observation of PGP as an oxidative stress modifier was based on a protocol that necessitated weeks of intermittent exposure to sublethal H_2_O_2_ concentrations (*29*). To investigate whether PGP deletion renders cells resistant to lethal oxidative stress in an acute setting, we performed viability assays in HT1080 cells after overnight incubation with serial dilutions of H_2_O_2_. We found that the KO of *PGP* did not suppress the toxicity of exogenous H_2_O_2_ under these conditions (Fig. 3A). Likewise, PGP loss did not restrain the cancer cell cytotoxicity of the pan-thioredoxin reductase (TXNRD) inhibitor auranofin or the cytosolic TXNRD1 inhibitor TRi-1, which trigger intracellular H_2_O_2_ production (*41*). Furthermore, PGP loss did not prevent cell death triggered by the pro-oxidant menadione, which has recently been shown to suppress prostate cancer progression in mice (*42*), nor did it counteract cancer cell death triggered by the reactive oxygen species (ROS)–producing chemotherapeutics doxorubicin, etoposide, carboplatin, or sorafenib (Fig. 3A) (*43*, *44*). Thus, PGP ablation does not cause a general resistance to oxidative cell death.

While the agents tested above are powerful pro-oxidants, they can induce multiple alternate cell death pathways (including apoptosis, autophagy, necrosis, ferroptosis, or triaptosis) (*42*, *45*, *46*). Furthermore, acute oxidative insults can be effectively ameliorated by the export of peroxisomal catalase to the cytoplasm (*29*). To explore possible consequences of PGP deficiency for a type of lethal oxidative damage that is directly linked to an exhaustion of the GSH antioxidant system, we investigated whether PGP loss affects ferroptosis. A key antiferroptotic axis involves cystine uptake via xCT, NADPH-dependent reduction of cystine to the more soluble cysteine (*27*), cysteine-dependent GSH biosynthesis, and the NADPH/GSH/GPX4-dependent detoxification of lipid peroxides (Fig. 1). We therefore tested the effect of PGP loss on ferroptosis triggered with xCT or GPX4 inhibitors. Figure 3B shows that PGP deletion led to a mild protection against ferroptosis induced with the xCT inhibitor erastin, which might be explained by compensatory GSH production through the transsulfuration pathway (see Fig. 2, E and F). In contrast, PGP loss robustly prevented ferroptosis in cells treated with the covalent GPX4 inhibitors RSL3, ML162, or ML210. Likewise, pronounced ferroptosis protection was seen when *PGP*-KO cells were treated with FIN56, a small molecule that triggers GPX4 degradation and coenzyme Q10 depletion (Fig. 3B). Furthermore, PGP deletion prevented cellular lipid peroxidation under ferroptotic stress, as indicated by reduced fluorescence of oxidized C11-BODIPY (581/591) in cells treated with FIN56, ML162, or ML210 (Fig. 3C).

To assess whether PGP deficiency affects ferroptosis in other cell types, we deleted *Pgp* by CRISPR-Cas9 in Hepa 1-6 murine hepatocellular carcinoma cells or used RNA interference to transiently or stably silence *PGP*/*Pgp* in HeLa (human cervical carcinoma) or GC1-spg (murine spermatogonial) cells (*47*), respectively. As shown in Fig. 3D, *Pgp* KO or *PGP* knockdown dampened ferroptosis in Hepa 1-6 or HeLa cells, respectively (fig. S1D), whereas *Pgp* knockdown in GC1-spg cells did not. These results show that PGP deficiency can confer ferroptosis protection in a cell type–dependent manner. More work in other cancer cell models and in primary cells is necessary to define the determinants of PGP-dependent ferroptosis suppression.

### Effects of PGP loss on cellular lipids

**Fig. 4.**
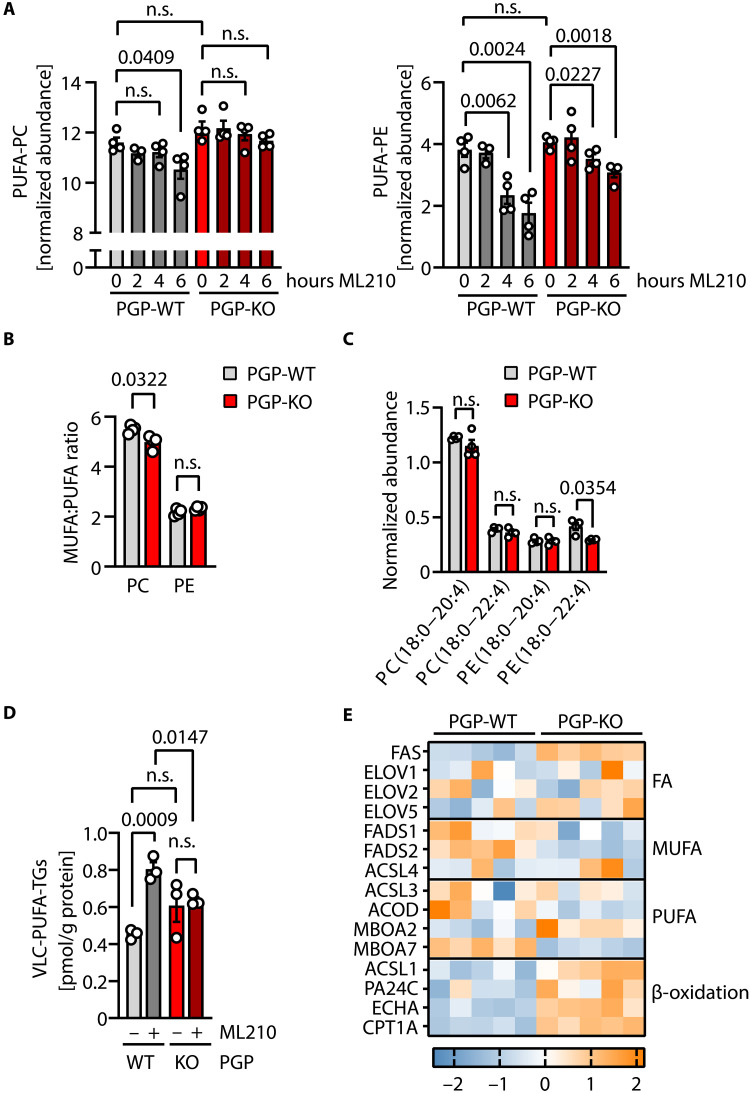
Effects of PGP deletion on cellular lipids. *PGP*-WT or *PGP*-KO HT1080 cells were treated with ML210 (0.5 μM) or the DMSO solvent control (0.1%, v/v) for the indicated times, and cellular lipids were analyzed by mass spectrometry. (**A**) Analysis of the PUFA content of PC and PE PLs. (**B**) Analysis of the MUFA:PUFA ratio of PC and PE PLs. (**C**) Abundance of PC and PE species with the indicated fatty acid tails. (A to C) All data are mean values ± SE of *n* = 4 biologically independent experiments. (**D**) Analysis of the abundance of very-long-chain (VLC) PUFA-TGs in *PGP*-WT or *PGP*-KO cells treated with ML210 (0.5 μM) or the DMSO solvent control (0.1%, v/v) for 2.5 hours. Data are mean values ± SE of *n* = 3 biologically independent experiments. Statistical analysis in (A) to (D): Unpaired, two-sided *t* tests; *P* values are indicated. (**E**) Heatmap of baseline expression changes in lipogenic enzymes (FA, fatty acid) and of enzymes involved in β-oxidation in *PGP*-WT and *PGP*-KO HT1080 cells. The *z*-scores of mean values in *n* = 5 biologically independent proteomic experiments are indicated. n.s., not significant.

PGP activity has been implicated in triglyceride (TG) biosynthesis (*38*), and we have previously found elevated levels of long-chain, polyunsaturated fatty acids (PUFAs) in *Pgp*-inactivated mouse embryos (*48*) and in *Pgp*-deficient mouse spermatogonial cells (*47*). To examine whether changes in lipid composition contribute to the observed ferroptosis resistance of PGP-deficient cells, we treated *PGP*-WT and *PGP*-KO HT1080 cells with ML210 and analyzed cellular lipids by mass spectrometry (fig. S2, A and B). In *PGP*-WT cells, ML210 treatment decreased the PUFA content of phosphatidylcholine (PC) and phosphatidylethanolamine (PE) species in a time-dependent manner (Fig. 4A), consistent with an increased cleavage of oxidatively damaged and truncated PUFAs from PC and PE phospholipids (PLs) in cells under ferroptotic stress. In contrast, PGP deletion prevented or dampened the ML210-induced decrease in PUFA-PCs or PUFA-PEs, respectively (Fig. 4A).

While PUFAs are particularly prone to peroxidation, monounsaturated fatty acids (MUFAs) are less oxidizable, and an increased MUFA:PUFA ratio can promote a ferroptosis-resistant state (*49*). However, we found that the PC-MUFA:PUFA ratio was lower in *PGP*-KO than in *PGP*-WT cells, whereas the PE-MUFA:PUFA ratio was similar in both genotypes (Fig. 4B). We therefore next explored the cellular PUFA composition. PUFAs that contain bisallylic protons, such as arachidonic acid (20:4, ω-6) and adrenic acid (22:4, ω-6), are highly susceptible to hydrogen atom abstraction (*7*, *50*). The abundance of PE(18:0-22:4) species was significantly reduced in *PGP*-KO compared to *PGP*-WT cells (Fig. 4C). This finding raises the possibility that PGP deficiency prevents ferroptosis in part by lowering the levels of PE species with adrenoyl tails.

We have previously shown that PGP deletion in HT1080 cells increases the abundance of lipid droplets (*35*), which are known to prevent ferroptosis by sequestering oxidatively damaged PUFAs away from the membrane as PUFA-TGs (*51*). However, we found that the pharmacological inhibition of diacylglycerol acyltransferase 1/2 (DGAT1/2; the enzymes that catalyze the committed step of TG synthesis) did not impede the ferroptosis protection of *PGP*-KO cells, although lipid droplet formation under baseline conditions was blocked (fig. S2C). In addition, although the detected PUFA-TGs (fig. S2B) tended to be more abundant in *PGP*-KO cells under baseline conditions, ML210 treatment significantly elevated the levels of PUFA-TGs in *PGP*-WT, but not in *PGP*-KO cells (Fig. 4D), consistent with the increased resistance of PUFA PLs to ferroptotic stress in the absence of PGP (Fig. 4A). To further explore the mechanisms underlying these changes, we performed a whole-cell proteomic analysis of *PGP*-WT and *PGP*-KO HT1080 cells, and first examined lipogenic enzymes involved in fatty acid, MUFA or PUFA biosynthesis, or metabolism. We observed an elevated expression of fatty acid synthase and of very long chain fatty acid elongase 5/ELOVL5, together with changes in membrane-bound glycerophospholipid *O*-acyltransferase 2 and 7 (MBOA2/7) expression, indicating altered lipid biosynthesis and PL remodeling (Fig. 4E). An altered membrane composition may be key to understand why PGP deficiency can specifically confer ferroptosis protection, without preventing cell death induced by pro-oxidants in general (Fig. 3). Levels of key proteins involved in mitochondrial β-oxidation were also clearly elevated in *PGP*-KO cells (Fig. 4E), pointing to changes in mitochondrial energy metabolism in the absence of PGP.

### PGP loss increases NRF2 levels and alters the expression of ferroptosis regulators

**Fig. 5.**
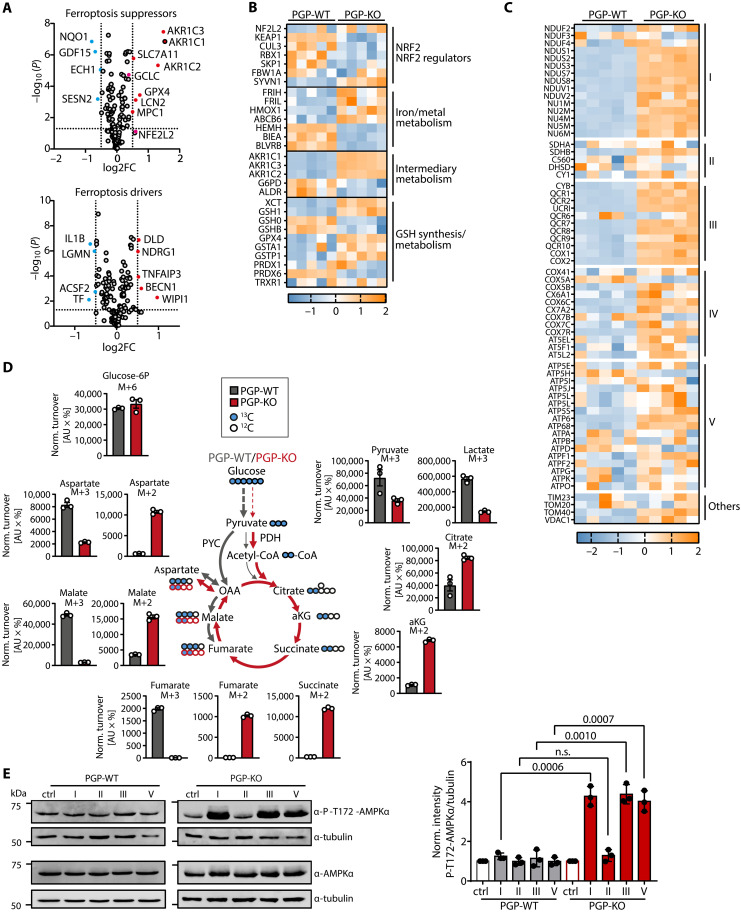
Effect of PGP deletion in HT1080 cells on ferroptosis regulators and mitochondrial metabolism. (**A**) Analysis of differentially abundant ferroptosis suppressors and ferroptosis drivers in the whole-cell proteomes of *PGP*-KO versus *PGP*-WT cells. (**B**) Heatmap of baseline expression changes (*z*-scores) in NRF2, NRF2 regulators, and ferroptosis-related NRF2-targets in *PGP*-WT and *PGP*-KO cells. (**C**) Heatmap of baseline expression changes (*z*-scores) in main components of the mitochondrial ETC complexes I to IV in *PGP*-WT and *PGP*-KO cells. TIM23, TOM20/40, and VDAC1 are shown for comparison. All data in (A) to (C) are mean values from *n* = 5 biologically independent proteomic experiments. (**D**) Isotopic tracing experiments. *PGP*-WT or *PGP*-KO cells were incubated with ^13^C_6_-labeled glucose for 30 min and analyzed by mass spectrometry. The normalized turnover values (peak area × percentage of labeling) of the indicated metabolites are shown. All data are means ± SE of *n* = 3 biologically independent experiments. OAA, oxaloacetate; aKG, α-ketoglutarate; PYC, pyruvate carboxylase; PDH, pyruvate dehydrogenase. (**E**) Left: Analysis of Thr^172^-P-AMPK and AMPK levels in *PGP*-WT or *PGP*-KO cells treated for 30 min with DMSO [0.1% (v/v), ctrl] or with the following ETC inhibitors: complex I, 5 μM piericidin A; complex II, 100 μM TTFA; complex III, 5 μM antimycin A; complex V, 5 μM oligomycin. The same lysates were probed with either Thr^172^-P-AMPK or AMPK antibodies, and tubulin was used as a loading control on the same membrane. Representative Western blots are shown. Right: Summary of *n* = 3 biologically independent experiments; mean values ± SE are shown. Statistical analysis: Unpaired, two-sided *t* tests; *P* values are indicated. AU, arbitrary units; FC, fold-change.

We next examined the expression of 502 known ferroptosis suppressors and drivers (*52*) in the whole-cell proteomes of *PGP*-WT and *PGP*-KO HT1080 cells. By focusing first on the most highly and significantly altered proteins, we found that PGP loss resulted in an up-regulation of ferroptosis suppressors including GPX4, solute carrier family 7 member 11 (SLC7A11), the iron-sequestering protein lipocalin-2 [LCN2; (*53*)], and the NADPH-dependent aldo-keto reductase family 1 members C1, C2, and C3 (AKR1C1-3), and in a down-regulation of ferroptosis drivers such as legumain [involved in autophagic GPX4 degradation; (*54*)] and transferrin [TF; (*7*)] (Fig. 5A and fig. S3A).

We also noted that the expression of NRF2, a master regulator of cellular redox homeostasis and ferroptosis (*55*), tended to be higher in *PGP*-KO cells (Fig. 5A). Inspection of NRF2 regulators and targets in our proteomic dataset revealed that the NRF2-inhibitory Kelch-like ECH-associated protein 1/Cullin 3/RING-box protein 1 (KEAP1/CUL3/RBX1) E3 ubiquitin ligase complex was down-regulated in *PGP*-KO cells, consistent with elevated NRF2 levels (Fig. 5B). Furthermore, some antiferroptotic NRF2 targets in iron metabolism [such as ferritin heavy chain (FRIH) and ferritin light chain (FRIL), which store iron in a nontoxic form], in intermediary metabolism (AKR1C1-3), and in GSH biosynthesis and metabolism [SLC7A11, GPX4, glutamate-cysteine ligase catalytic subunit/GSH1, glutathione *S*-transferase A1 (GSTA1), and glutathione *S*-transferase P1 (GSTP1)] were enriched in *PGP*-KO cells, whereas others were decreased (Fig. 5B). AKR1C1-3 can detoxify reactive aldehydes generated downstream of PUFA oxidation, and AKR1C1-3 up-regulation has been implicated in partial resistance to ferroptosis induction by xCT inhibition (*56*). Nevertheless, S07-2010, a potent pharmacological pan-AKR1C inhibitor [half-maximal inhibitory concentration (IC_50_) values between 0.2 and 0.7 μM for AKR1C1-4 isoforms; (*57*)] was insufficient to compromise the ferroptosis protection afforded by PGP deletion in our system (fig. S3B). Together, these data indicate that PGP loss triggers a battery of cytoprotective responses, including increased GSH biosynthesis, increased detoxification of reactive lipid metabolites, diminished iron-induced oxidative damage, and altered lipid metabolism (Fig. 4), which might jointly contribute to the observed ferroptosis resistance.

NRF2 abundance is a tightly regulated, stress-inducible transcription factor, and different E3-ubiquitin ligase complexes can control NRF2 levels (*55*). In HT1080 cells, PGP deficiency was associated with a depletion of the KEAP1/CUL3/RBX1 complex under baseline conditions (Fig. 5B). To elucidate possible mechanisms underlying the altered expression of KEAP1/NRF2 and of ferroptosis regulators in PGP-deficient cells, we performed a biological pathway enrichment analysis of *PGP*-WT and *PGP*-KO HT1080 whole-cell proteomes. *PGP*-KO cells were highly enriched in proteins involved in the mitochondrial electron transport chain (ETC) and in oxidative phosphorylation (OXPHOS) (Fig. 5C and fig. S3C), suggesting an up-regulation of mitochondrial respiration in *PGP*-KO cells.

Mass spectrometry–based quantitation of citric acid (TCA) cycle metabolites together with isotopic tracing using ^13^C_6_-labeled glucose showed a preferred turnover of pyruvate-derived, M + 3–labeled aspartate and of the oxaloacetate-derived TCA cycle metabolites malate and fumarate in *PGP*-WT cells (Fig. 5D). This is consistent with a slowing down of TCA cycle activity when cells have ample reduced form of nicotinamide adenine dinucleotide (NADH) and adenosine 5′-triphosphate (ATP) (*58*). In contrast, PGP loss led to a strong increase in the turnover of acetyl-coenzyme A (CoA)–derived, M + 2–labeled TCA cycle metabolites (Fig. 5D), reflecting higher activity (fig. S3D) of TCA cycle enzymes in *PGP*-KO cells. Together with the comparable abundance and labeling of glucose 6-P in *PGP*-WT and *PGP*-KO cells, and the reduced turnover of both pyruvate and lactate in *PGP*-KO cells (Fig. 5D), these data clearly demonstrate that PGP loss causes a glycolytic block, which triggers an increase in TCA cycle activity.

To functionally test the importance of increased TCA cycle activity in *PGP*-KO cells, we measured the Thr^172^ phosphorylation levels of the cellular energy sensor adenosine 5′-monophosphate (AMP)–activated kinase alpha (AMPKα) as a readout for AMPK activation (*59*). As shown in Fig. 5E, Thr^172^-P-AMPK levels in *PGP*-WT and *PGP*-KO cells were comparable under baseline conditions, whereas treatment with ETC complex I, III, or V inhibitors strongly increased Thr^172^-P-AMPK levels in *PGP*-KO, but not in *PGP*-WT cells. Treatment with the complex II inhibitor 2–thenoyltrifluoroacetone (TTFA) only affected P-Thr^172^-AMPK levels when this compound was used at very high concentrations (fig. S3E). Thus, comparable Thr^172^-P-AMPK phosphorylation levels under baseline conditions in *PGP*-WT and *PGP*-KO cells indicate that the reduced glycolytic activity of PGP-deficient cells is compensated for by increased TCA cycle and OXPHOS activity. In contrast, uncoupling the TCA cycle activity from the ETC and OXPHOS by blocking ETC complexes leads to energy stress in *PGP*-KO cells.

The mitochondrial ETC is an important source of cellular ROS (*60*), and mitochondrial ROS and TCA metabolites can trigger KEAP1 degradation and activate a cytoprotective response network via NRF2 (*61*). The increased TCA cycle activity in *PGP*-KO cells may be accompanied by elevated ROS generation and KEAP1 degradation and could explain how PGP loss is linked to NRF2 activation and ferroptosis suppression.

### The PGP inhibitor CP1 has PGP-independent, ferroptosis-sensitizing effects

The genetic inactivation of *Pgp* is embryonically lethal due to a block in proliferation (*48*). Similarly, CRISPR-Cas9–mediated PGP deletion leads to a strong proliferation defect, although cells can adapt and resume proliferation over time (*35*). For these reasons, and because siRNAs have been reported to commonly sensitize cells to ferroptosis (*62*), we have analyzed only a limited number of cell lines and no nonimmortalized or primary cells for PGP-dependent effects on ferroptosis so far (Fig. 3).

**Fig. 6.**
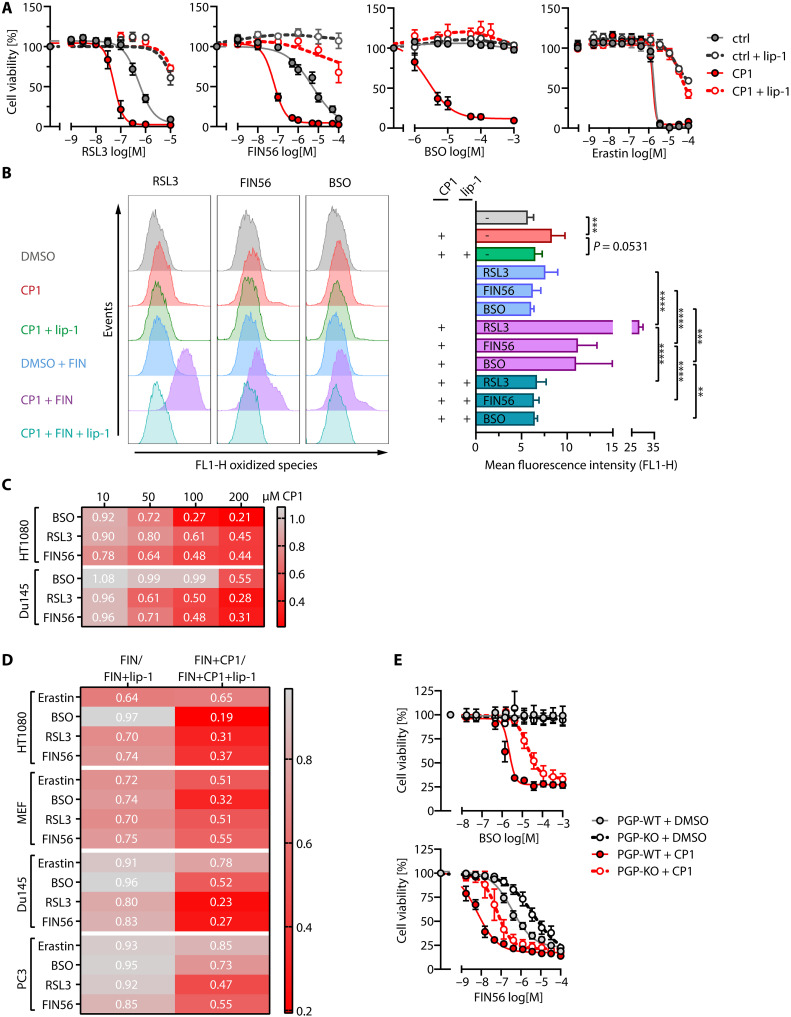
Effect of CP1 on cellular ferroptosis sensitivity. (**A**) HT1080 cells were incubated with the indicated ferroptosis inducers (FINs) in the absence (DMSO control) or presence of CP1 and/or the ferroptosis inhibitor lip-1. (**B**) Effect of CP1 on cellular lipid peroxidation investigated with C11-BODIPY (581/591). HT1080 cells were treated with the DMSO solvent control, CP1, or lip-1 in the presence of sublethal concentrations of RSL3 (50 nM), FIN56 (50 nM), or BSO (1 μM). Left: Representative flow cytometry analysis. Right: Summary of *n* = 4 (RSL3/FIN56) or *n* = 3 (BSO) experiments; data are means ± SD. Statistical testing: One-way analysis of variance (ANOVA) and Tukey’s multiple comparisons test, ****P < 0.0001; ***P < 0.001; **P < 0.01. (**C**) Concentration dependency of CP1 effect. Cells were treated with the indicated FINs ± CP1 ± lip-1. Concentration-response curves were constructed by nonlinear regression, *n* = 3. Area under the curve values were calculated and are given in the heatplot. (**D**) Area under the curve metrics of cell line ferroptosis sensitivity to CP1. Cells were treated with the indicated FINs ± CP1 ± lip-1 (HT1080, Du145, PC3: 200 μM CP1, MEF: 100 μM CP1). Area under the curve values were calculated as in (C); *n* ≥ 3. (**E**) *PGP*-WT and *PGP*-KO HT1080 cells were treated with the indicated FINs ± CP1. Data are means ± SE, *n* = 3. [(A) to (E)] All incubations were for 16 hours. DMSO solvent control (0.1%, v/v), CP1 (100 μM), lip-1 (100 nM), unless otherwise stated. Cell viability was measured with AquaBluer. Apparently missing error bars are hidden by the symbols. For all experiments, *n* refers to the number of biologically independent experiments.

We therefore considered a pharmacological approach to inhibit PGP. Using a combination of small-molecule screening, protein crystallography, molecular dynamics simulations, and nuclear magnetic resonance–based metabolomics, we have recently identified and characterized the first experimental PGP inhibitor, termed CP1 (*35*). Although CP1 is relatively potent in vitro (with an IC_50_ value of 0.4 μM for human and mouse PGP), high CP1 concentrations (100 μM) are required to reach sufficient intracellular concentrations of this compound (*35*). To test whether ferroptosis can be suppressed by inhibiting PGP pharmacologically, we thus treated HT1080 cells with 100 μM CP1. Unexpectedly, in contrast to the expected ferroptosis protection due to PGP inhibition, we found that CP1 strongly sensitized HT1080 cells to RSL3- or FIN56-induced cell death, and CP1-potentiated cell death was entirely prevented by the ferroptosis inhibitor liproxstatin-1 (Fig. 6A). We verified that CP1 treatment alone did not substantially decrease cell viability under these conditions (fig. S4A). Buthionine sulfoximine (BSO), a glutamate-cysteine ligase inhibitor that blocks GSH biosynthesis, did not induce ferroptosis by itself, but concentration-dependently triggered massive cell death in the presence of CP1 (Fig. 6A). Ferroptosis resulting from impaired GSH biosynthesis downstream of xCT inhibition with erastin was not enhanced by CP1 (Fig. 6A).

Flow cytometry analyses with the C11-BODIPY (581/591) lipid peroxidation sensor showed that CP1 treatment by itself slightly enhanced C11-BODIPY fluorescence. In the presence of sublethal concentrations of RSL3, FIN56, or BSO, CP1 robustly augmented C11-BODIPY fluorescence in a liproxstatin-1–dependent manner (Fig. 6B). Hence, CP1 renders cells more vulnerable to lipid peroxidation resulting from compromised GPX4 activity.

The ferroptosis-sensitizing effect of CP1 was concentration-dependent (Fig. 6C) and observed not only in other human cancer cells lines such as Du145 and PC3 prostate carcinoma cells but also in primary mouse embryonic fibroblasts (MEFs; Fig. 6D). PGP overexpression did not alter the cellular response to CP1 in the presence of GPX4 inhibitors (fig. S4B), and the treatment of *PGP*-KO cells with CP1 in combination with GPX4 inhibitors attenuated the ferroptosis-suppressing effect of PGP deletion (Fig. 6E). The levels of the key ferroptosis regulators GPX4, FSP1, and acyl-CoA synthetase long-chain family member 4 (ACSL4) were unaffected by CP1 (fig. S4C; see also Fig. 4E), and CP1 did not potentiate HT1080 or PC3 cell death when combined with eight different cytotoxic agents that can trigger cell death through various, predominantly ferroptosis-independent mechanisms (fig. S4, D and E). In sum, these unexpected results suggest that CP1 sensitizes human and rodent cells to ferroptotic cell death in a PGP-independent manner.

### CP1 is a direct inhibitor of human and murine FSP1

**Fig. 7.**
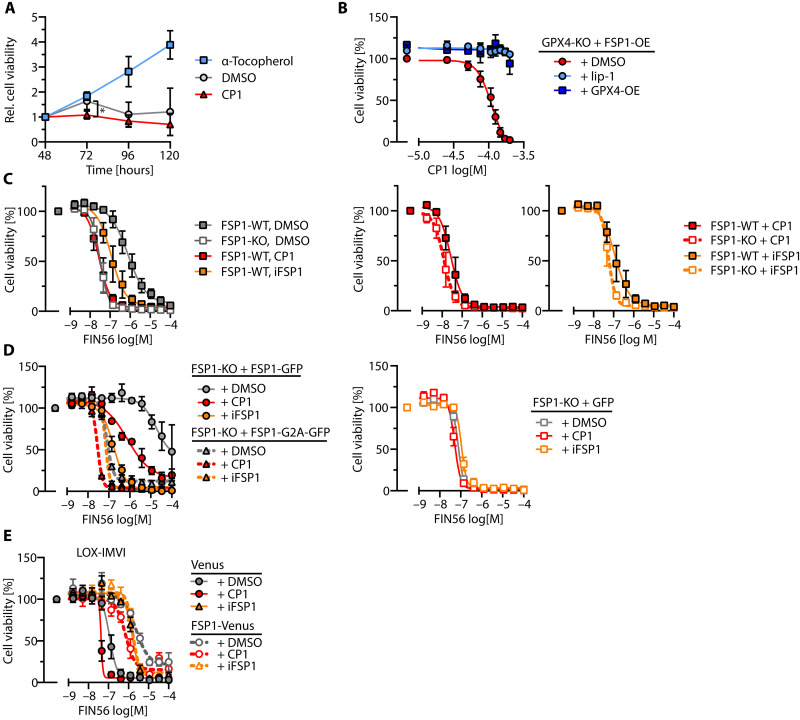
Role of FSP1 for CP1-induced ferroptosis sensitization. (**A**) HT1080 *GPX4*-KO cells were grown with α-tocopherol, or α-tocopherol was washed out at *t* = 0, and cells were maintained in the absence (DMSO solvent control) or presence of CP1 over five consecutive days. Statistical analysis: Paired, two-sided *t* test, *P = 0.0385. (**B**) Effect of CP1 on ferroptosis in FSP1-dependent [*GPX4*-KO + *FSP1*-overexpression (OE)] or FSP1-independent (*GPX4*-KO + *FSP1*-OE + *GPX4*-OE) HT1080 cells incubated with serial dilutions of CP1 in the absence of other ferroptosis inducers. (**C**) Left: Comparison of CP1 and iFSP1 on FIN56-induced ferroptosis in HT1080 *FSP1*-WT or *FSP1*-KO cells. Middle or right: *FSP1*-WT or *FSP1*-KO cells treated with CP1 or iFSP1. The curves of inhibitor-treated *FSP1*-WT cells are identical to the respective curves in the left panel. (**D**) Role of FSP1 membrane targeting for CP1 or iFSP1-induced ferroptosis sensitization. Left: HT1080 *FSP1*-KO cells reconstituted with FSP1-WT or *FSP1*- $\mathrm{Gly2}\;\to \;\mathrm{Ala}\left({\mathrm{FSP1}}^{\mathrm{G2A}}\right)$. Right: Comparison of CP1 or iFSP1 effects in *FSP1*-KO control cells expressing GFP. (**E**) Comparison of CP1 and iFSP1 effects on ferroptosis sensitivity in LOX-IMVI cells. [(B) to (E)] All data are means ± SE of *n* ≥ 3 biologically independent experiments. Cell viability was assessed with AquaBluer after 16-hour incubation with the indicated compounds. DMSO solvent control (0.1% v/v), α-tocopherol (100 μM), CP1 (100 μM), iFSP1 (10 μM), lip-1 (100 nM). Apparently missing error bars are hidden by the symbols.

We sought to further elucidate the mechanisms underlying this unexpected finding. Because CP1 strongly increased the cytotoxicity of three mechanistically distinct GPX4 inhibitors (see Fig. 6A), we first asked whether CP1 facilitates ferroptotic cell death in a GPX4-independent manner. For this, HT1080 *GPX4*-KO cells (fig. S5A) maintained in the presence of α-tocopherol were subjected to α-tocopherol washout and then cultured in the absence or presence of CP1. This approach shows that CP1 significantly accelerates ferroptosis in the absence of GPX4 (Fig. 7A).

The survival of *GPX4*-KO cells has been demonstrated to depend on FSP1 (*16*, *17*). We found that *GPX4*-KO cells overexpressing FSP1 died in a CP1 concentration–dependent manner, whereas GPX4 add-back or liproxstatin-1 treatment rendered them CP1-insensitive (Fig. 7B and fig. S5B). Furthermore, the comparison of *FSP1*-WT or *FSP1*-KO cells cultured in the presence of GPX4 inhibitors revealed that the extent of ferroptosis sensitization by CP1 overlapped with the effect of FSP1 deletion, similar to the established FSP1 inhibitor iFSP1 (Fig. 7C and fig. S5C) (*17*). Like iFSP1, CP1 only sensitized cells expressing membrane binding–competent FSP1 to ferroptosis (Fig. 7D and fig. S5, D and E). We also observed that CP1 did not further enhance ferroptosis in naturally FSP1-deficient LOX-IMVI cells (a BRAF-$\mathrm{VaL600}\;\to \;\mathrm{Glu}\left({\mathrm{BRAF}}^{\mathrm{V600E}}\right)$ heterozygous human melanoma cell line) and that FSP1 overexpression in these cells antagonized the CP1 effect (Fig. 7E and fig. S5F). As described previously, iFSP1 was ineffective in the LOX-IMVI cell model (*17*). Together, these results clearly point to FSP1 as a candidate CP1 target.

**Fig. 8.**
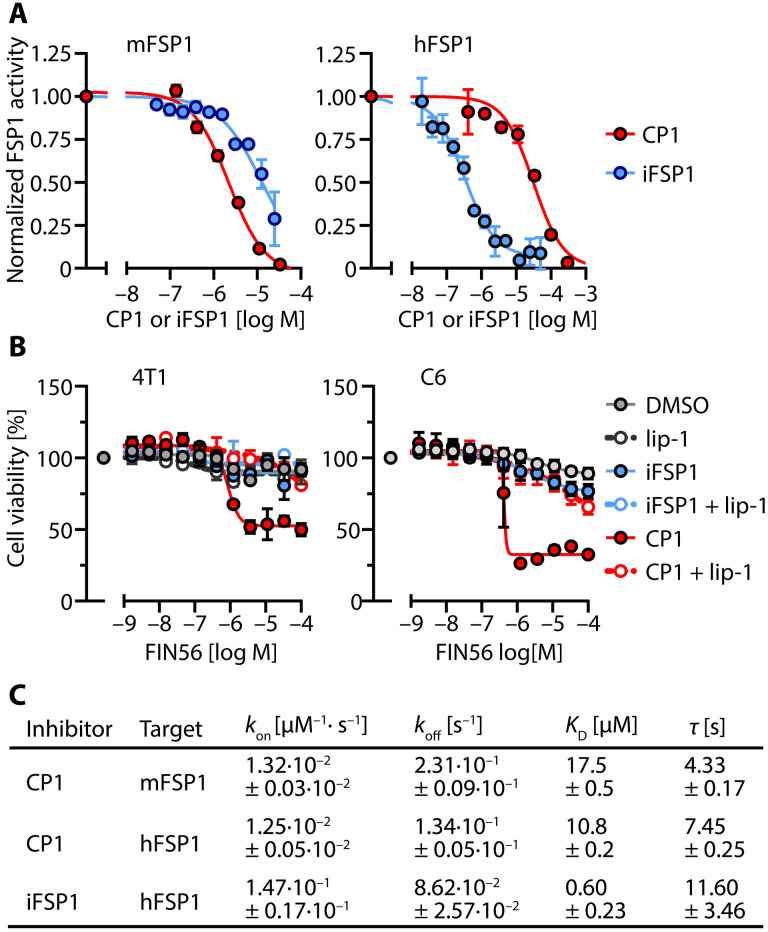
CP1 is a direct inhibitor of hFSP1 and mFSP1. (**A**) Determination of CP1 or iFSP1 IC_50_. Shown are NADH consumption assays using recombinant, purified mFSP1 or hFSP1 and resazurin as an electron acceptor. FSP1 oxidoreductase activities in the presence of CP1 or iFSP1 were normalized to the respective enzyme activities measured in the presence of the solvent control DMSO (0.1%, v/v). (**B**) 4T1 mouse breast carcinoma or C6 rat glioblastoma cells were incubated for 16 hours with serial dilutions of FIN56 in the absence [0.1% (v/v) DMSO solvent control] or presence of CP1 (100 μM), iFSP1 (10 μM), or lip-1 (100 nM). Cell viability was analyzed with AquaBluer. (A and B) Data are means ± SE of *n* = 3 biologically independent experiments. Apparently missing error bars are hidden by the symbols. (**C**) BLI measurements of the interaction of CP1 with recombinant, purified, biotinylated Avi-tagged mFSP1 or hFSP1, or of iFSP1 with hFSP1. Summary data are means ± SE of *n* = 4 technically independent measurements. τ, residence time.

To test this directly, we conducted in vitro activity assays with recombinant, purified FSP1. CP1 directly inhibited the enzymatic activity of human and murine FSP1 (hFSP1 and mFSP1), as measured by NADH oxidation using resazurin as an electron acceptor. Compared to iFSP1, CP1 is a more potent mFSP1 inhibitor (IC_50_ CP1 = 2.2 ± 0.3 μM; IC_50_ iFSP1 = 12.9 ± 2.0 μM), but a less potent hFSP1 inhibitor (IC_50_ CP1 = 31.1 ± 4.5 μM; IC_50_ iFSP1 = 0.33 ± 0.07 μM) (all data are mean values ± SE of *n* = 3 biologically independent experiments) (Fig. 8A). Thus, CP1 inhibits FSP1 in a similar concentration range as PGP [CP1 IC_50_ for PGP = 0.4 μM; (*35*)], and the CP1 concentrations used in cellular assays may suffice to inhibit FSP1. Consistent with reports that iFSP1 inhibits only hFSP1 in cells (*17*), we observed that iFSP1 did not sensitize 4T1 mouse breast carcinoma and C6 rat glioblastoma cells to FIN56-induced ferroptosis, whereas CP1 was active in these rodent cells (Fig. 8B).

We next used a biolayer interferometry (BLI) optical biosensing technique to characterize the binding of CP1 and iFSP1 to mFSP1 or hFSP1. Consistent with a specific interaction, CP1 binding to both mFSP1 and hFSP1 was concentration-dependent and fully reversible (fig. S6). Global analysis of the sensorgrams of a CP1 serial dilution series assuming a 1:1 binding model resulted in an affinity (*K*_D_) value of 17.5 ± 0.5 μM for mFSP1 and 10.8 ± 0.2 μM for hFSP1 (Fig. 8C). Whereas the association rate constant (*k*_on_) of CP1 for mFSP1 and hFSP1 was comparable, the dissociation rate constant (*k*_off_) of CP1 was twofold higher for mFSP1 than for hFSP1, resulting in a longer residence time of CP1 on hFSP1 (Fig. 8C). The *k*_on_ of iFSP1 for hFSP1 was about 10-fold higher than for CP1. Together with a slightly lower *k*_off_, this resulted in a longer residence time and a 17-fold lower *K*_D_ of iFSP1 for hFSP1 compared to CP1. The binding of iFSP1 to mFSP1 was not detectable by BLI. Together, these data establish CP1 as a direct inhibitor of mFSP1 and hFSP1, both in vitro and in cells.

In our previous work, we identified two other candidate PGP inhibitors (the CP1-related compound CP2, and a structurally more distinct molecule termed CP3) and characterized a small panel of commercially available CP1 analogs (*35*). We next profiled the selectivity of these compounds for PGP and FSP1 (fig. S7). We found that CP2 is 5.5-fold more PGP-selective than CP1 and that CP3 is a PGP inhibitor that lacks detectable activity against mFSP1. We also observed that the CP1 fragment CP-C5 preferentially inhibits mFSP1 compared to mPGP (fig. S7). Together, these data demonstrate that PGP- and FSP1-inhibitory activities of this class of compounds can be separated and that CP-C5 may represent a lead structure for the development of FSP1 inhibitors.

Although potent pharmacological hFSP1 inhibitors have been identified (*17*, *63*–*65*), only one compound (viFSP1) has been reported so far that is also able to block mFSP1 (*65*). All published FSP1 inhibitors are structurally unrelated to CP1. CP1 therefore represents a molecule with a previously uncharacterized FSP1-inhibitory chemical scaffold. Because the discovery of species-independent FSP1 inhibitors is important for preclinical studies and drug development, we decided to further explore the properties of CP1 as an FSP1 inhibitor.

### CP1 likely targets the FSP1 NAD(P)H binding pocket

Recent work has shown that iFSP1 targets the quinone binding pocket and is coordinated by amino acid residues that are present in human, but not murine or rat FSP1, thus explaining the species-selectivity of this inhibitor (*65*, *66*). Similarly, FSEN1 is a potent inhibitor of hFSP1 that is unable to block mFSP1 (*63*). FSEN1 targets the hFSP1 substrate-binding pocket and makes key interactions with Phe^360^, which is replaced by a Leu^360^ in mFSP1, explaining the FSEN1 selectivity for hFSP1 (*67*). In contrast, viFSP1, a species-independent FSP1 inhibitor, targets the NAD(P)H binding pocket (*64*). Based on our findings so far, we hypothesized that CP1 might also address the FSP1 NAD(P)H binding site.

FSP1 has two conserved NAD(P)H-binding motifs, GxGxxGxE and WxxG, and one conserved flavin adenine dinucleotide (FAD)–binding motif (GD). Except for the ${\mathrm{Glu}}^{156}\;\to \;\mathrm{Ala}\left(E156\;A\right)$ mutant, which retains minimal oxidoreductase function, mutations of the other conserved amino acids in the NAD(P)H- and FAD-binding motifs abrogate FSP1 enzymatic activity (fig. S8A) (*16*, *65*). We tested the effect of CP1 on recombinantly expressed, purified mFSP1^E156A^ and found that this mutant was approximately sevenfold less sensitive to CP1 inhibition than mFSP1-WT (fig. S8B). These data suggest that Glu^156^ in the NAD(P)H binding pocket of FSP1 is involved in CP1 binding.

Global docking of CP1 to the hFSP1-NADP complex [Protein Data Bank (PDB): 8JSC] indicates that the CP1 and NADP binding sites overlap (fig. S8C). The primary CP1/FSP1 interaction site is the FSP1 region that binds the nicotinamide moiety of NADP, near Glu^156^. CP1 binding is mediated by one of its two 2-aminobenzoic acid groups (see fig. S7A). Like NADP, CP1 forms a hydrogen bridge to Lys^293^.

We next examined the effect of CP1 on FSP1 steady-state kinetics, using NADH consumption assays and resazurin as an artificial substrate (*17*, *65*). When the cosubstrate NADH was nonlimiting and resazurin concentrations were increased, CP1 reduced both the Michaelis-Menten constant (*K*_M_) and the maximal velocity (*v*_max_) of hFSP1 and mFSP1. This indicates an uncompetitive binding mode, where the inhibitor binds to the enzyme-substrate complex. However, when resazurin was nonlimiting and NADH concentrations were increased, CP1 had little effect on *v*_max_, but increased the *K*_M_ of hFSP1 and mFSP1, indicating a competitive mode of inhibition. These data suggest that CP1 may compete with NAD(P)H binding to FSP1 (table S2). To directly analyze this further, we used BLI to measure the binding of NADH to mFSP1 in the absence or presence of CP1. Steady-state analysis of an NADH serial dilution series yielded an NADH binding affinity (*K*_D_) value of 820 μM for mFSP1. However, in the presence of a saturating concentration of CP1 (50 μM), NADH binding to mFSP1 was no longer detectable (fig. S9). These results further support an NAD(P)H-competitive binding mode of CP1. Unfortunately, our attempts to cocrystallize CP1 and FSP1 to definitively prove CP1 binding in the NAD(P)H pocket have not been successful thus far.

### CP1 enhances FSP1 dimer and oligomer formation in vitro

**Fig. 9.**
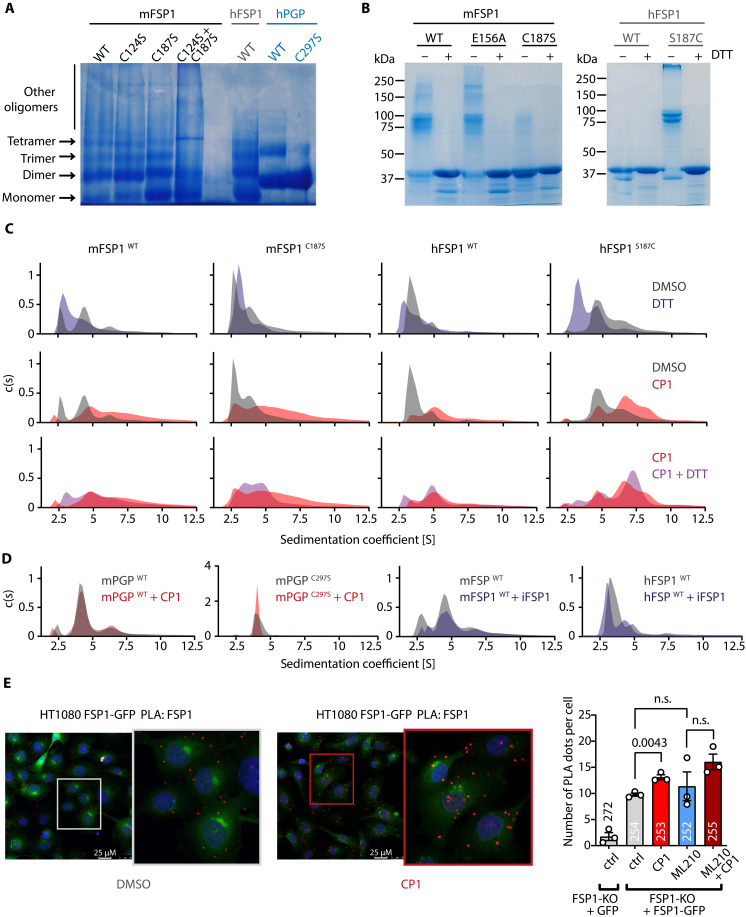
Effects of CP1 on FSP1 self-assembly. FSP1 oligomerization profile analyzed by (**A**) BN-PAGE (20 μg protein/lane, 4 to 13% acrylamide gradient gel) or (**B**) nonreducing PAGE (10 μg protein, preincubated ± 5 mM DTT, 8% SDS-PAGE gels). FSP1 molecular mass, 41 kDa. Size references: Human PGP-WT, 34 kDa, forming homodimers and homotetramers, and the homotetramerization-incompetent PGP- ${\mathrm{CYS}}^{297}\;\to \;\mathrm{Ser}\left({\mathrm{PGP}}^{\mathrm{C297S}}\right)$ mutant (*35*, *74*). (**C**) AUC analyses. FSP1 proteins were incubated with the DMSO solvent control (0.2%, v/v), CP1 (100 μM), and/or DTT (5 mM). Data are mean values of *n* = 3 biologically independent experiments, except for hFSP1^S187C^ + DTT and hFSP1^S187C^ + CP1 + DTT (*n* = 2 biologically independent experiments). Peak sedimentation coefficients of FSP1 monomers: ~2.5 *S*; FSP1 dimers: ~4.5 *S*. (**D**) AUC analyses of mPGP-WT or mPGP^C297S^ ± CP1 (100 μM) and mFSP1-WT or hFSP1-WT ± iFSP1 (100 μM). Data are means of *n* = 3 biologically independent experiments. Peak sedimentation coefficients of PGP dimers: ~4 *S*; PGP tetramers: ~6.5 *S*. See fig. S11 for histograms of all AUC experiments. (**E**) PLA. The indicated HT1080 cells were treated with DMSO (0.1%, v/v) or CP1 (100 μM) for 16 hours, followed by an incubation with DMSO (0.1%, v/v) or ML210 (0.5 μM) for 1.5 hours. Cells were fixed, and FSP1 self-assembly was detected as detailed in Materials and Methods. Left: Representative fluorescence micrograph. Scale bars, 25 μM. Right: Summary data. On average, ~85 cells were scored per experiment and per condition; the total number of scored cells is indicated above or in the bars. Data are mean values ± SE of *n* = 3 biologically independent experiments. Statistical analysis: Unpaired, two-sided *t* tests; *P* values are indicated.

An inhibitor of hFSP1 (icFSP1) has recently been shown to induce phase separation of FSP1 into molecular condensates and to promote ferroptosis (*64*), suggesting that self-assembly and sequestration of FSP1 away from membranes is an important determinant of FSP1 function. However, little is known about possible oligomeric states of FSP1 orthologs and their regulation. Using blue native polyacrylamide gel electrophoresis (BN-PAGE), we observed that recombinant, purified mFSP1 and hFSP1 can exist as mono-, di-, and oligomeric species. The major mFSP1 species is a dimer, whereas hFSP1 is predominantly monomeric and dimeric (Fig. 9A). hFSP1 and mFSP1 differ in the presence of a surface-exposed Ser or Cys residue in position 187, respectively (fig. S10, A and B) (*68*). Exchanging Cys^187^ for Ser in mFSP1 increased the monomer fraction and resulted in an hFSP1-like oligomerization profile. The exchange of Cys^124^ for $\mathrm{Ser}\;\mathrm{in}\;\mathrm{mFSP1}\left({\mathrm{mFSP1}}^{\mathrm{C1245}}\right)$ did not affect oligomerization and was therefore not studied further (Fig. 9A). The analysis of the FSP1 oligomerization profile with nonreducing gels demonstrated that mFSP1 and hFSP1 differ in their tendency to oligomerize, that FSP1 self-assembly is reversible, and that a Cys or a Ser residue in position 187 determines redox-dependent FSP1 oligomerization (Fig. 9B and fig. S10). In addition, a Ser or Cys residue in FSP1 position 187 affects CP1 sensitivity (fig. S10, C to E).

To investigate whether CP1 affects FSP1 homotypic interactions, we conducted analytical ultracentrifugation (AUC) measurements. To obtain information on FSP1 size distributions, we performed sedimentation velocity runs and determined sedimentation coefficients. In agreement with gel-based results (Fig. 9, A and B), we observed that mFSP1 is mostly dimeric, whereas mFSP1^C187S^ is mainly monomeric. hFSP1 is predominantly monomeric, and hFSP1^S187C^ exists as dimeric and oligomeric species. The mFSP1^E156A^ AUC profiles resembled the mFSP1-WT profiles (fig. S10F). Thus, a Cys or Ser residue in position 187 affects the intrinsic tendency of FSP1 for self-association. Reducing conditions led to a shift in the size distributions of mFSP1-WT and of hFSP1^S187C^ toward monomers, showing that FSP1 dimer and oligomer formation is reversible (rather than being an artefact caused by protein misfolding; Fig. 9C, top). CP1 diminished the fraction of FSP1 monomers and increased the dimers and the continuum of oligomeric species (Fig. 9C, middle). These data indicate that CP1 can drive and/or stabilize FSP1 self-assembly, irrespective of whether the major FSP1 species is monomeric or dimeric and irrespective of the presence of a Cys or Ser residue in FSP1 position 187. Reducing conditions also had little impact on the CP1-induced mFSP1 or hFSP1 self-assembly (Fig. 9C, bottom).

We verified that the effect of CP1 on FSP1 self-association is specific (Fig. 9D). PGP, the first identified CP1 target, is a homodimer that exists in a dynamic equilibrium with tetramers (*30*, *35*). However, CP1 neither affected the PGP dimer/tetramer ratio nor triggered self-assembly of the tetramerization-incompetent PGP^C297S^ mutant (*34*, *57*). Furthermore, the FSP1 inhibitor iFSP1 had no effect on mFSP1 or hFSP1 oligomerization (Fig. 9D). Together, these in vitro results show that CP1 can specifically enhance FSP1 self-assembly. While the presence of a Ser or Cys residue in position 187 affects the redox-dependent, intrinsic tendency of purified FSP1 to self-associate (and the sensitivity of FSP1 to CP1-mediated enzymatic inhibition; see table S2 and fig. S10), CP1-induced FSP1 self-assembly is largely redox-independent.

Last, we asked whether CP1-induced FSP1 self-assembly can be observed in cells. To visualize homotypic FSP1 interactions, we conducted proximity ligation assays (PLAs). This method has been successfully used to detect homotypic protein-protein interactions in cells (*69*). We treated HT1080 *FSP1*-KO cells stably expressing green fluorescent protein (GFP) or FSP1-GFP (fig. S5E) with the dimethyl sulfoxide (DMSO) solvent control, CP1, and/or ML210 and detected FSP1 self-assembly with a mouse monoclonal α-FSP1 antibody linked to Plus or Minus oligonucleotides. We then visualized the PLA signals by fluorescence microscopy. PLA signals were visible as red fluorescent dots and readily detectable in FSP1-GFP–expressing cells, whereas only few PLA signals were visible in the FSP1-deficient GFP control cells, indicating that the method is specific (Fig. 9E). CP1 led to a significant increase in the number of PLA dots per cell, whereas ML210 treatment alone did not (Fig. 9E). These results suggest that CP1 can enhance FSP1 self-assembly in a cellular environment. Nevertheless, whether these PLA signals reflect FSP1 homodimers or larger oligomers and whether they contribute to FSP1 sequestration and hence to a FSP1 loss of function remains to be further investigated.

## DISCUSSION

PGP deletion has been shown to mitigate long-term oxidative stress, but the underlying mechanisms remained unexplored (*29*). Here, we investigate the consequences of PGP deletion on acute oxidative insults and find that PGP deletion suppresses ferroptosis but does not prevent oxidative cell death in general. We find that PGP loss alters the membrane composition and triggers a battery of cytoprotective responses, including increased GSH biosynthesis, altered lipid metabolism, and an increased capacity to detoxify reactive lipid metabolites and to control iron-induced oxidative damage.

Mechanistically, we demonstrate that PGP deletion in HT1080 cells triggers two widely used cellular antioxidant defense mechanisms. First, we show that the absence of PGP reroutes glucose catabolism into the oxPPP, via the accumulation of the PGP substrate 4PE and the subsequent inhibition of 6PGDH. This can fuel NADPH production and GSH-mediated cellular antioxidant defense capacity and explain the seemingly paradoxical observation that silencing the core oxPPP enzyme 6PGDH protects against ferroptosis (*14*). Previous work from other groups has established that a disruption of the PPP at the level of 6PGDH protects cancer cells subjected to long-term oxidative stress by metabolic rewiring (*29*). An immediate redirection of glucose flux into the oxPPP is seen in human skin fibroblasts and keratinocytes exposed to oxidants, potentially maximizing NADPH-dependent ROS clearance (*70*, *71*). In addition, activated primary human neutrophils rapidly switch from a glycolysis-dominant metabolism to a cyclic form of the PPP, where all glucose 6-phosphate is diverted into the oxPPP, maximizing NADPH yield to power NADPH oxidase activity and pathogen elimination (*72*). Second, we delineate a pathway where cellular energy metabolism in PGP-deficient cells is maintained by elevated mitochondrial TCA cycle and OXPHOS activity to compensate for the block in glycolytic pathway flux. Increased mitochondrial oxidative stress under these conditions could relieve the KEAP1-mediated repression of NRF2 and in part explain the plethora of cytoprotective and antiferroptotic responses observed upon PGP deletion. Energy stress caused by PGP deficiency may also inhibit ferroptosis via AMPK activation and the associated changes in membrane composition (*73*).

Our previous work has shown that the activity and oligomerization state of PGP is controlled by reversible oxidation, both in vitro and in cells subjected to induced oxidative stress by stimulation with H_2_O_2_ or EGF (*74*). Hence, PGP can be a relevant cellular target of reversible oxidation. Specifically, we identified four cysteine residues that are necessary and sufficient to relay the reversible oxidation of PGP: three cysteine residues in the PGP catalytic core domain that mediate the reversible PGP inhibition (Cys^35^, Cys^104^, and Cys^243^) and one cysteine residue (Cys^297^) that is essential for redox-dependent PGP oligomerization (*74*). Our crystallography analyses further demonstrated that Cys^104^ and Cys^243^ can form a disulfide bridge that can interfere with the structural changes that enable the enzyme’s catalytic cycle (*32*, *35*, *74*). Our ongoing research aims to establish whether PGP can act as a physiological redox sensor that enables cells to respond to oxidative stress with altered ferroptosis resistance.

Another important area of future study is to define the cell type specificity of ferroptosis suppression due to PGP loss, i.e., to elucidate whether cancer or immune cells become ferroptosis-resistant upon PGP targeting or, conversely, to examine which cell types may benefit from ferroptosis suppression via PGP inhibition. Given the multitude of metabolic factors that control the balance between cellular protection and ferroptotic demise and the complex rewiring of intermediary metabolism triggered by PGP deletion, it is likely that the consequences of PGP targeting are context dependent. Highly glycolytic cells may be particularly responsive to PGP inhibition and ferroptosis suppression, possibly also via energy stress–mediated AMPK activation (*73*).

To address these questions, selective pharmacological PGP inhibitors would be important tools. Unexpectedly, our attempts to block PGP with CP1, the first described PGP inhibitor that we identified recently (*35*), led to our finding that CP1 is in fact a dual PGP and FSP1 inhibitor. This dual activity may be linked to the presence of Rossmann-fold domains in both PGP and FSP1, although CP1 did not substantially inhibit 12 other phosphatases harboring a Rossmannoid fold (*35*). The discovery of CP1 as an FSP1 inhibitor adds another chemical scaffold to the growing toolbox of ferroptosis sensitizers. CP1 has interesting features that could be further refined in future drug development efforts. Like viFSP1 (*65*), CP1 directly inhibits both mFSP1 and hFSP1, a prerequisite for preclinical drug testing. Thus far, only the human-specific FSP1 inhibitor icFSP1 has been reported to induce FSP1 phase-separation in cells, although the exact mechanism is elusive (*64*). We demonstrate here that CP1 can directly trigger FSP1 self-association, both in vitro and in cells. It remains an open question whether CP1-induced FSP1 self-assembly is involved in sequestering FSP1 away from membranes and whether this is linked to FSP1 inhibition by phase separation and the formation of molecular condensates (*64*). We note that FSP1 oligomerization per se does not lead to an inhibition of FSP1 oxidoreductase activity, as clearly demonstrated by the comparable oxidoreductase activity of monomeric hFSP1 and dimeric/oligomeric hFSP1^S187C^. The identification of the CP1 binding sites that mediate FSP1 self-assembly might facilitate the development of improved inhibitors to further study and exploit this intriguing mechanism for cancer therapy.

## MATERIALS AND METHODS

### Consumables

Unless otherwise specified, all reagents were of the highest available purity and purchased from Sigma-Aldrich. FIN56, iFSP1, ML210, ML162, and S07210 were from MedChemExpress, and C11-BODIPY 581/591 was from Molecular Probes/Thermo Fisher Scientific. CP1, CP1 analogs, CP2, and CP3 were obtained from ChemDiv.

### Antibodies

Antibodies were purchased from the following providers: Sigma-Aldrich (α-actin: mAb1501, RRID: AB_2223041), Cell Signaling Technology (α-tubulin: DM1A, no. 3873, RRID: AB_1904178; α-GAPDH: 14C10, no. 2118, RRID: AB_561053; α-AMPKα: D63G4, no. 5832, RRID: AB_10624867; α-phospho-Thr^172^-AMPKα: 40H9, no. 2535, RRID: AB_331250), Santa Cruz Biotechnology (α-GPX4: E-12, sc-166570, RRID: AB_2112427; α-PGP: E10, sc-390883, RRID: AB_3719354; α-FSP1: E1, sc-376987, RRID: AB_3705475; α-ACSL4: A-5, sc-271800, RRID: AB_10715092), Proteintech (α-perilipin-3: no. 10694-1-AP, RRID: AB_2297252; α-AIFM2/FSP1: 1A2B2, no. 68049-1-PBS, RRID: AB_3719355), and Molecular Probes/Thermo Fisher Scientific (Alexa Fluor 488–labeled goat anti-rabbit secondary antibodies).

### Molecular cloning

For BLI experiments, codon-optimized hFSP1 (UniProt Q9BRQ8) and mFSP1 (UniProt Q8BUE4) (*17*) were cloned into the Nco I and Eco RI restriction sites of pETM11. The AviTag peptide (GLNDIFEAQKIEWHE) was inserted at the respective N termini (i.e., C terminus of the His_6_-tag and the TEV cleavage site in pETM11), using inverse PCR mutagenesis as described previously (*75*). cDNAs were amplified using Q5 Hot Start High-Fidelity DNA Polymerase (NEB). Similarly, mFSP1-C124S was cloned into pETM11. All other constructs were in pET-SUMO, using Platinum SuperFi II (Thermo Fisher Scientific) and the following primers (oligonucleotide sequence 5′-3′; fwd, forward; rev, reverse) for mutagenesis: *hFSP1-S187C*, fwd TACTGCCGTGCGTTCGTCA and rev ATTCCTTGTCCGCCAGCGC; *mFsp1-C124S*, fwd AATGAAGTTTCATCTCAGCAAGCCCA and rev TGCGGCTTGCTGAGATGAAACTTCATT; *mFsp1-C187S*, fwd GAGCTCCTGCCATCTGTCCGACAAGAA and rev TTCTTGTCGGACAGATGGCAGGAGCTC; and *mFsp1-E156A*, fwd TCTGCTGGTGTGGCGATGGCTGCTGAGATA and rev TATCTCAGCAGCCATCGCCACACCAGCAGA. All constructs were verified by sequencing.

### Cell culture, CRISPR-Cas9, RNA interference, and transfection

HT-1080, PC3, Du145, HeLa, MDA-MB-231, 4T1, and C6 cells (all from LGC Standards) were cultured in Dulbecco’s modified Eagle’s medium [DMEM; glucose (4.5 g/liter)] supplemented with 10% (v/v) heat-inactivated fetal calf serum (FCS; Anprotech), 2 mM l-glutamine, and penicillin (100 U/ml) and streptomycin (100 μg/ml) (Pen/Strep) (“complete medium”). LOX-IMVI (BRAF-V600E heterozygous human melanoma) cells (*17*) were grown in RPMI-1640 supplemented with 10% FCS, 2 mM l-glutamine, and Pen/Strep. Cell culture media and supplements were from PAN-Biotech. Du145, Hepa 1-6, or 4T1 cells were gifts from H. Hermanns and E. Henke, respectively. GPX4 and/or FSP1-targeted HT1080 cells and LOX-IMVI cells were described previously (*17*). Nontransformed, nonimmortalized MEFs were generated from *Pgp*-WT (*Pgp^fl/fl^*) or heterozygous *Pgp*-inactivated mouse embryos (*Pgp^WT/D34N^*) as described (*48*).

Single-cell HT1080 *PGP*-WT and *PGP*-KO clones (*PGP*-WT, clone KE6; *PGP*-KO, clone A3B7) were generated using CRISPR-Cas9 as described previously (*35*), using the human GeCKOv2 library single guide RNA HGLibA_36277 (5′-CAACCCCGAGCGCACCGTCA-3′ in pLentiCRISPRv2) or the empty control vector (pLentiCRISPRv2). Single-cell *Pgp*-WT and *Pgp*-KO Hepa 1-6 cells were generated using CRISPR-Cas9 as above, using the murine CHOPCHOP v3 library and the target sequence 5′-GCTCGAACCCGACGTGCGCGCGG-3′ (*76*). For RNA interference studies, HeLa cells were transiently transfected with siRNA oligonucleotides targeting *Pgp* mRNA or with control siRNA as described (*30*), using the PepMute siRNA transfection reagent according to the manufacturer’s recommendations (SignaGen Laboratories). For PGP overexpression, parental HT1080 cells were transfected with Lipofectamine 2000 (Invitrogen).

### Western blotting

Cultured cells were lysed in buffer A [50 mM tris (pH 7.5), 150 mM NaCl, 1% (v/v) Triton X-100, 0.5% (w/v) sodium deoxycholate, 0.1% (w/v) sodium dodecyl sulfate, aprotinin (5 μg/ml), leupeptin (1 μg/ml), pepstatin (1 μg/ml), and 1 mM 4-(2-aminoethyl)benzenesulfonyl fluoride (Pefabloc)]. For the analysis of AMPK Thr^172^ phosphorylation, HT1080 *PGP*-WT or *PGP*-KO cells (100,000 cells per 35-mm dish) were incubated overnight and treated with the indicated ETC inhibitors (5 μM piericidin A, 5 μM antimycin A, 5 μM oligomycin, and 100 μM TTFA) or the DMSO solvent control for 30 min at 37°C in a tissue culture incubator. Cells were lysed in buffer A supplemented with phosphatase inhibitors [1% (v/v) phosphatase inhibitor cocktail 2 and 1% (v/v) phosphatase inhibitor cocktail 3] and 10 mM sodium pyrophosphate. Protein concentrations were determined using the Micro BCA Protein Assay Kit (Thermo Fisher Scientific). Proteins were separated by SDS-PAGE, transferred to nitrocellulose membranes by semidry-blotting, and incubated with the indicated antibodies. Western blots were quantified using NIH Image (https://imagej.net/nih-image/).

### Cell viability assays

Cells were seeded onto 96-well plates (5000 cells per well). The next day, they were treated for 16 hours with serial dilutions of ferroptosis-inducing compounds or cytotoxic drugs in the absence or presence of 100 μM CP1 or 10 μM iFSP1 (unless stated otherwise) or the DMSO solvent control. Ferroptotic cell death was evaluated by incubating in the absence or presence of liproxstatin-1 (100 nM), ferrostatin-1 (200 nM), or deferoxamine (100 μM); apoptosis was assessed with the pan-caspase inhibitor zVAD-FMK (100 μM). Cell viability was assessed after 16 hours using resazurin (Cayman Chemical) or AquaBluer (MoBiTec), according to the manufacturer’s recommendations. Fluorescence (excitation, 540 nm; emission, 590 nm) was analyzed on a CLARIOstar microplate reader (BMG Labtech).

### Assessment of lipid peroxidation with flow cytometry

Approximately 10,000 HT1080 *PGP*-WT or *PGP*-KO cells were seeded per well of a 48-well dish. The next day, cells were treated for 1 hour with DMSO (0.1%, v/v), FIN56 (5 μM), ML210 (0.3 μM), or ML162 (0.25 μM) in the absence or presence of liproxstatin-1 (100 nM). Cells were incubated with 1 μM C11-BODIPY (581/591) for 30 min at 37°C in a tissue culture incubator, harvested by trypsinization, and resuspended in 150 μl of phosphate-buffered saline (PBS) supplemented with soybean trypsin inhibitor (0.5 mg/ml) and 1% FCS. Cells were analyzed using the 488-nm laser of a flow cytometer (MACSQuant Analyzer 16, Miltenyi Biotec) for excitation. Data were collected from the B1 detector with a 525/50 nm band-pass filter for oxidized species, or from the B2 detector with a 585/40 nm band-pass filter for nonoxidized species. At least 5000 cells were analyzed per sample. The C11-BODIPY (581/591) oxidation ratio was calculated for each condition.

To analyze the effect of CP1, HT1080 cells were seeded as described above. The next day, cells were treated for 16 hours with DMSO (0.1%, v/v), CP1 (100 μM), or liproxstatin-1 (100 nM) in the absence or presence of sublethal concentrations of RSL3 (50 nM), FIN56 (50 nM), or BSO (1 μM). Cells were incubated with C11-BODIPY (581/591), harvested as described above, and analyzed using the 488-nm laser of a FACSCalibur flow cytometer (BD Biosciences) for excitation. Data were collected from the FL1 detector with a 530 nm band-pass filter. At least 5000 cells were analyzed per sample. All flow cytometry data were analyzed using FlowJo.

### Lipid droplet staining

*PGP*-WT or *PGP*-KO HT1080 cells were seeded on glass coverslips (10,000 cells per well of a 12-well plate) in complete DMEM. The next day, cells were incubated for 16 hours with 10 μM of the DGAT1 inhibitor T863 and 5 μM of the DGAT2 inhibitor PF-06424439 or with 0.1% (v/v) of the DMSO solvent control. Cells were fixed for 10 min with 4% (w/v) paraformaldehyde (PFA), permeabilized and blocked with 0.2% (w/v) saponin/2% (w/v) bovine serum albumin (BSA) in PBS for 1 hour, and immunostained for 1 hour using α-rabbit perilipin 3 antibody [1:200 dilution in 1% (w/v) BSA/PBS]. Primary antibodies were detected with Alexa Fluor 488–labeled goat anti-rabbit secondary antibodies (1:400), and nuclei were counterstained with 4′,6-diamidino-2-phenylindole (DAPI). Cells were mounted with Immu-Mount (Thermo Fisher Scientific) and imaged by fluorescence microscopy on a THUNDER imager (THUNDER Imager Live Cell and 3D Cell Culture and 3D Assay System, Leica DMI8, Leica Microsystems), using a 40× objective and the Leica Application Suite X software 3.7.4.23463. To determine the number of lipid droplets per cell, cell borders were defined semiautomatically, and bright objects in this region of interest were counted using ImagePro-Plus v7.0 (Media Cybernetics).

### Proximity ligation assays

PLA probes to detect FSP1 were prepared according to the instructions in the PLA probe maker kit, by linking the α-AIFM2/FSP1 mouse monoclonal 1A2B2 antibody (in PBS) with either Plus or Minus oligonucleotides (Millipore DUO92009 or DUO92010, respectively). HT1080-*FSP1*-KO cells expressing either GFP (control) or FSP1-GFP (see fig. S6E) were seeded on fibronectin (10 μg/ml)–coated 12-well ibidi chamber slides (ibidi no. 81201; 10,000 cells per well). The following day, cells were treated with DMSO (0.1%, v/v) or CP1 (100 μM) for 18 hours. Afterward, cells were incubated with or without 0.5 μM ML210 for 1.5 hours, fixed with 4% (w/v) PFA in PBS for 20 min, and permeabilized with 0.25% (v/v) Triton X-100 in PBS for 10 min. Blocking, ligation, amplification, and detection steps were then performed according to the manufacturer’s protocol (Duolink PLA Fluorescence Detection Kit, red DUO94001, Millipore). Briefly, after blocking, cells were incubated for 1 hour at room temperature (RT) with the PLA Plus and Minus probes. Nuclei were stained with DAPI. Cells were imaged on a Keyence BZ-X800 microscope with a 40× objective and on a Leica DMI6000 confocal microscope with a 63× objective and analyzed using ImagePro-Plus v7.0.

### Mass spectrometry

#### Analysis of water-soluble metabolites

HT1080 *PGP*-WT or *PGP*-KO cells were cultured as described above, seeded in 10-cm dishes, and harvested the next day (~1 million cells per dish). For this, medium was removed, and the cells were rinsed twice with ice-cold ammonium acetate (154 mM). Cells were lysed in 0.9 ml of ice-cold MeOH/H_2_O (80/20, v/v), scraped into Eppendorf tubes and snap-frozen in liquid nitrogen. Water-soluble metabolites were extracted and subjected to mass spectrometry analysis as described (*35*).

For isotopic tracing experiments, *PGP*-WT or *PGP*-KO HT1080 cells were seeded at a density of 0.5 million cells per 10-cm dish in complete DMEM containing 4.5 g glucose/liter. Twenty-two hours later, the medium was replaced by 1.5 ml of complete DMEM containing U-^13^C_6_-glucose (4.5 g/liter; Cambridge Isotope Laboratories no. CLM-1396-2, 99% ^13^C). After incubation for 0, 15, 30, and 60 min at 37°C and 5% CO_2_ [time 0 corresponds to a medium replacement with complete DMEM containing unlabeled glucose (4.5 g/liter) for 1 hour], the cells were washed twice with 5 ml of ice-cold ammonium acetate (154 mM) and lysed in 0.9 ml of MeOH/H_2_O (80/20, v/v). Samples were flash-frozen in liquid nitrogen and kept at −80°C until analysis.

#### Lipidomics

HT1080 *PGP*-WT or *PGP*-KO cells were cultured, seeded, and washed as described above. Cells were treated for the indicated time points with ML210 (0.5 μM) or DMSO (0.1%, v/v). For harvesting, cells were washed as described above, scraped into 0.8 ml of ice-cold MeOH/H_2_O (50/50, v/v), transferred into Eppendorf tubes, and frozen at −80°C. Lipid extraction, analysis, and data mining were performed as previously described (*48*).

#### Tandem-Mass-Tag proteomics

HT1080 *PGP*-WT or *PGP*-KO cells were cultured as described above, seeded in 15-cm dishes, and grown overnight. The next day, *PGP*-WT or *PGP*-KO cells were washed once with PBS and lysed at RT in lysis buffer [8 M urea, 50 mM tris (pH 8.4), and 150 mM NaCl, containing freshly added phosphatase inhibitors (5 mM sodium molybdate, 5 mM sodium fluoride, and 10 mM sodium β-glycerophosphate) and EDTA-free protease inhibitor cocktail (Roche Life Sciences)]. Cells were then scraped into Eppendorf tubes and snap-frozen in liquid nitrogen. Lysates were sonicated and centrifuged (15,000*g* for 30 min at 4°C), and supernatants were transferred to a new tube. The protein concentration was determined using a BCA assay (Pierce/Thermo Fisher Scientific). Dithiothreitol (DTT) and iodoacetamide were added to reduce and alkylate, respectively. Proteins were precipitated with methanol and chloroform and resuspended in 8 M urea and 50 mM Hepes (pH 8.5). Urea was diluted 6× with 50 mM Hepes (pH 8.5), prior to an overnight incubation at 37°C with 1:100 (w/w) trypsin. The next day, the trypsin digest was stopped by the addition of 0.25% trifluoroacetic acid (TFA) (final v/v). Peptides were desalted over an Oasis HLB plate (Waters).

Peptides were labeled with Tandem-Mass-Tag (proTMT) reagent (Thermo Fisher Scientific). Once labeling efficiency was confirmed to be at least 95%, each reaction was quenched by adding hydroxylamine to a final concentration of 0.25% for 10 min and then mixed, acidified with TFA to a pH of ~2, and desalted over an Oasis HLB plate. The desalted multiplex was dried by vacuum centrifugation and separated by offline pentafluorophenyl-based reversed-phase high-performance liquid chromatography fractionation as previously described (*77*). TMT-labeled peptides were analyzed on an Orbitrap Lumos mass spectrometer (Thermo Fisher Scientific) equipped with a Vanquish Neo liquid chromatography system (Thermo Fisher Scientific). In brief, samples were analyzed on the Orbitrap Lumos operating in data-dependent, SPS-MS3 quantification mode wherein an Orbitrap MS1 scan was taken [scan range = 350 to 1250 mass/charge ratio (*m*/*z*), *R* = 120 K, AGC target = 2.5 × 10^5^, maximum ion injection time = 50 ms], followed by data-dependent ion trap MS2 scans on the most abundant precursors for 1.8 s. Ion trap MS2 parameters were as follows: ion selection charge state = 2: minimum intensity 2 × 10^5^ and precursor selection range 600 to 1400, ion selection charge state = 3 to 5: minimum intensity 3 × 10^5^; quadrupole isolation = 0.8 *m*/*z*, collision-induced dissociation (CID) collision energy = 35%, CID activation time = 10 ms, activation Q = 0.25, scan range mode = auto, AGC target = 4 × 10^3^, maximum ion injection time = 40 ms. Parameters for Orbitrap MS3 scans for quantification were as follows: resolution = 50 k, AGC target = 5 × 10^4^, maximum ion injection time = 100 ms, higher-energy collisional dissociation collision energy = 45%, scan range = 100 to 500 *m*/*z*, synchronous precursors selected = 6 (*78*).

The raw data files were searched using COMET with a static mass of 229.162932 Da on peptide N termini and lysines and 57.02146 Da on cysteines and a variable mass of 15.99491 Da on methionines against the target-decoy version of the human proteome sequence database (UniProt; downloaded February 2013, 40,482 entries of forward and reverse protein sequences), maximum of three missed cleavages allowed, precursor ion mass tolerance of 1 Da, and fragment ion mass tolerance of ±8 parts per million, and filtered to a <1% false discovery rate at the peptide level. Quantification of liquid chromatography–tandem mass spectrometry spectra was performed using in-house developed software. Peptide intensities were adjusted based on total TMT reporter ion intensity in each channel and log_2_ transformed. *P* values were calculated using a two-tailed Student’s *t* test, assuming unequal variance.

#### Proteomic analyses

Validated, screened, predicted, or deduced ferroptosis regulators were retrieved from the ferroptosis database FerrDBv2 (www.zhounan.org/ferrdb) (*52*). After the removal of duplicates, all listed ferroptosis drivers or suppressors (264 or 238 proteins, respectively) were matched against the whole-cell proteomic dataset of HT1080 *PGP*-WT and *PGP*-KO cells. Hits were extracted, and their relative abundances were compared. Pathway enrichment/overrepresentation analysis was conducted with the WEB-based GEne SeT AnaLysis Toolkit (www.webgestalt.org). For this, the top 100 up-regulated proteins in *PGP*-KO cells were subjected to an overrepresentation analysis against the total proteome of HT1080 *PGP*-WT cells, using the GeneOntology database.

### Expression and purification of recombinant PGP and FSP1

Murine PGP and murine PGP^C297S^ were expressed and purified as described (*35*). His_6_-SUMO-tagged hFSP1 and murine FSP1^C187S^ were grown in ZYP-5052 autoinduction medium for 7 hours at 37°C, followed by 48 hours at 21°C (*79*). All other His_6_-SUMO-tagged FSP1 proteins were grown in LB medium for 20 hours at 20°C after induction with 0.2 mM isopropyl β-d-1-thiogalactopyranoside. All purification steps of FSP1 were carried out exactly as described for PGP (*35*), except that human SenP2 protease was used to cleave the His_6_-SUMO-tag. Protein concentrations were determined with the Micro BCA Protein Assay Kit.

### Biotinylation of Avi-tagged FSP1

Purified, AviTag-fused mFSP1 or hFSP1 were enzymatically biotinylated with the *Escherichia coli* biotin ligase BirA. To this end, FSP1 proteins (100 μM) were incubated with purified GST-BirA (a gift from P. Hänzelmann) in the presence of ATP and d-biotin, as described (*75*). GST-BirA was removed using GSH Sepharose 4 Fast Flow (GE Healthcare) followed by size exclusion chromatography.

### Biolayer interferometry

Biotinylated, AviTag-fused mFSP1 or hFSP1 (200 μg/ml) were equilibrated in BLI buffer [TMN buffer supplemented with 0.005% (v/v) Tween 20], loaded on streptavidin-coated biosensors (Super Streptavidin/SSA Dip and Read Biosensors, ForteBio), quenched with 27 μM biocytin, and washed in BLI buffer. Reference SSA sensors were blocked with 27 μM biocytin for 1 min. The SSA sensors were loaded to a shift of up to 16 nm with mFSP1-WT or 25 nm with hFSP-WT, respectively. Concentration-response titrations with CP1 and iFSP1 were performed using 1:1 serial dilution steps ranging from 25 to 0.39 μM for CP1 and 10 to 0.16 μM for iFSP1. Every concentration was measured four times. The final DMSO concentration was 0.1% (v/v) for CP1 and 0.33% (v/v) for iFSP1. Concentration-response titrations with NADH were performed using 1:1 serial dilution steps ranging from 5000 to 78.13 μM NADH, either in the absence or presence of CP1, kept at a constant concentration of 50 μM. The final DMSO concentration in all samples was 0.1% (v/v). Every concentration was measured three times. Buffers for baseline, dissociation, and buffer correction wells were supplemented with the same amount of DMSO for identical buffer conditions. The assay settings were as follows: baseline measurement of 60 s, association time of 150 s, and dissociation time of 240 s. All BLI measurements were performed on an Octet RED96e (Sartorius) device at 24°C. Kinetic analyses were performed in Origin Pro, using the double reference method of the Octet analysis software for removal of drifts and well-to-well artifacts. For the fitting, all measured data points of *n* = 4 technically independent measurements were considered for CP1. Due to the poor signal-to-noise ratio, the lowest iFSP1 concentration (0.16 μM) was not included in the analysis. For the fitting of NADH with or without CP1, the double referenced data were further analyzed in GraphPad Prism to estimate the affinity by fitting the signals of the steady states to a 1:1 Langmuir model. For the fitting, all measured data points of *n* = 3 technically independent measurements were considered.

### FSP1 oxidoreductase assays, IC_50_ determinations, and enzyme kinetics

NADH consumption assays were performed using recombinant, purified mFSP1 or hFSP1, and resazurin as an electron acceptor. For this, FSP1 proteins were diluted to the indicated final concentrations (3 μM mFSP1-WT; 0.3 μM for all other FSP1 proteins) in FSP1 assay buffer (375 mM NaCl and 100 mM Na_3_PO_4_, pH 7.0) and preincubated with the indicated compounds/inhibitors in the absence or presence of the indicated DTT concentrations for 10 min. NADH was added to a final concentration of 500 μM, and the absorbance at 340 nm (A_340_) was measured every 15 s at RT under shaking (300 rpm) on a CLARIOstar microplate reader. After a stable signal was reached, 200 μM resazurin was added to start the reaction. The first 10 measured values of the NADH consumption were used to calculate the slope with a linear regression function. The inversed, negative slope was used for further curve fitting and the calculation of IC_50_ values and Michaelis-Menten kinetics using GraphPad Prism 7.05. For IC_50_ determinations, log_inhibitor_ versus response was calculated.

Recombinant, purified PGP was preincubated for 15 min at RT with serial dilutions of 6PG. The dephosphorylation reaction was started by the addition of the indicated substrate; buffer without enzyme served as background control. Prior to testing, time courses of inorganic phosphate release from 6PG were conducted to ensure assay linearity. Inorganic phosphate release was detected with malachite green solution (Biomol Green, Enzo Life Sciences); the absorbance at 620 nm (*A*_620_) was measured on an Envision 2104 multilabel reader (Perkin Elmer Life Sciences). Released phosphate was determined by converting the *A*_620_ absorbance values to nmol inorganic phosphate (P_i_) with a phosphate standard curve. Data were analyzed with GraphPad Prism 7.05. For IC_50_ determinations, log _inhibitor_ versus response was calculated for a Hill slope of −1. To derive *K*_M_ and *v*_max_ values, data were fitted by nonlinear regression to the Michaelis-Menten equation.

### Docking

The crystal structure of the hFSP1 (PDB: 8JSC) was prepared for docking using the MOE (Molecular Operating Environment, 2026.03, Chemical Computing Group ULC, 1010 Sherbrooke St. West, Suite No. 910, Montreal, QC, Canada, H3A2R7, 2023) structure preparation tools with default values and was protonated at pH 7. For docking, all solvent atoms and the cofactor NADP were removed from the prepared complex structure. The ligand CP1 (prepared at pH 7) was docked using GOLD v2023.03. The binding site was defined by a sphere with a radius of 20 Å centered at Ile^245^. The ChemPLP scoring function was used for scoring, with a search efficiency of 200% being used (*80*). The internal_ligand_h_bond flag was set to on, and the COO^−^ groups were set to rotation mode. A set of 100 docking poses was generated and the top five poses with the highest rankings were visually validated by overlaying them with the NADP crystal structure of PDB: 8JSC.

### BN-PAGE and nonreducing PAGE

BN-PAGE was performed as published (*81*). Recombinant, purified proteins were diluted 1:10 to a final concentration of 20 μM in 50 mM triethanolamine and 5 mM MgCl_2_ (pH 7.4). Electrophoresis was conducted at 4°C in cathode buffer B [50 mM tricine, 7.5 mM imidazole, and 0.02% (w/v) Coomassie Blue G250 (pH 7.0)]. Once the running front had reached one third of the separating gel, cathode buffer B was replaced by cathode buffer B/10, containing 0.002% (w/v) Coomassie Blue G250. Gels were fixed for 30 min [50% methanol (v/v), 10% acetic acid (v/v), and 100 mM ammonium acetate], stained for 1 hour [0.025% Coomassie Blue G250 (w/v) in 10% acetic acid (v/v)], and destained in 10% acetic acid (v/v). Nonreducing SDS-PAGE was conducted using Laemmli buffer without β-mercaptoethanol.

### Analytical ultracentrifugation

Sedimentation velocity AUC was carried out using a Proteome Lab XL-I analytical ultracentrifuge (Beckman Coulter) with an eight-hole An-50 Ti rotor (Beckman Coulter). Four hundred microliters of purified recombinant protein, at a concentration of 10 μM in TMN1 buffer [250 mM NaCl, 50 mM triethanolamine, and 5 mM MgCl_2_ (pH 7.4)] containing 0.2% (v/v) DMSO, were loaded in one channel of standard double-sector charcoal-filled Epon centerpieces (Beckman Coulter) equipped with sapphire windows (Beckman Coulter), versus 400 μl of TMN1 + DMSO buffer reference in the second channel of the sample cell. Measurements were run in vacuum at 40,000 rpm and at 20°C with data collection in continuous mode every 5 min until sedimentation was complete (final exponential mass distribution at the bottom of the sample cell channel). Data detection was based on position and time-dependent sample density changes along the length of the sample cell channel resulting in refractive index differences, which were recorded using interference optics with a 660-nm diode laser. Data were analyzed using the NIH software SEDFIT v12.52 to determine continuous sedimentation coefficient distributions from numerical solutions to the Lamm equation that describes the movement of density boundaries of a sample species in the centrifugal field (*82*). Analysis was performed in a sedimentation coefficient range from 0 to 20 *S* with regularization at confidence levels of 0.68, time and position (radius) independent noise correction, and floating frictional ratio *f*/*f*_0_ to root mean square deviation values ≤0.02. Similar results were obtained with two independently purified protein batches and two independently synthesized CP1 batches. The area under the individual peaks in the sedimentation coefficient distribution curves indicates the population of the different oligomeric states. Areas were approximated for monomers, dimers, and higher-order oligomers using the trapezoidal rule (Eq. 1), where *x*_*k*/*k*−1_ are the sedimentation coefficient values and *f*(*x*_*k*/*k*−1_) are the corresponding c(s) function values. *x*_0_ (*x*_*k*−1_ for *k* = 1) and *x_N_* (*x_k_* for *k* = *N*) represent the lower and upper limits, respectively, of the total sedimentation coefficient range for which the area is calculated: 1.82 to 3.64 *S* for monomers, 3.64 to 5.86 *S* for dimers, and 5.86 to 15.15 *S* for oligomers

$$\int_{0}^{N}f\left(x\right)\mathrm{dx}≊\sum_{k=1}^{N}\left(x_{k}-x_{k-1}\right)\times \left[\frac{f\left(x_{k}\right)+f\left(x_{k-1}\right)}{2}\right]$$ (1)

### Statistical analysis and source data

The values for *n* independent experiments, measures of central tendency (e.g., mean or median), dispersion (e.g., SE and SEM), *P* values, and the specific statistical test performed for each experiment are included in the respective figure legends or the main text. If not indicated otherwise, data were analyzed using GraphPad Prism v10.6. Source data are given in the auxiliary table S3.
