# Supplementary material for: Metabolic rewiring driven by phosphoglycolate phosphatase deletion inhibits ferroptosis
Source: Sci Adv. 2026 May 29;12(22):eaeb2368. doi: 10.1126/sciadv.aeb2368 (PMC13220862; doi:10.1126/sciadv.aeb2368)
Supplement: Supplementary file 1 — Figs. S1 to S11 Tables S1 and S2 Legend for table S3 [file sciadv.aeb2368_sm.pdf]

Science Advances
AAAS

Supplementary Materials for

Metabolic rewiring driven by phosphoglycolate phosphatase deletion inhibits ferroptosis

Marian Brenner *et al.*

Corresponding author: Antje Gohla, antje.gohla@uni-wuerzburg.de

Sci. Adv. **12**, eaeb2368 (2026)
DOI: 10.1126/sciadv.aeb2368

The PDF file includes:

Figs. S1 to S11
Tables S1 and S2
Legend for table S3

Other Supplementary Material for this manuscript includes the following:

Table S3

Fig. S1

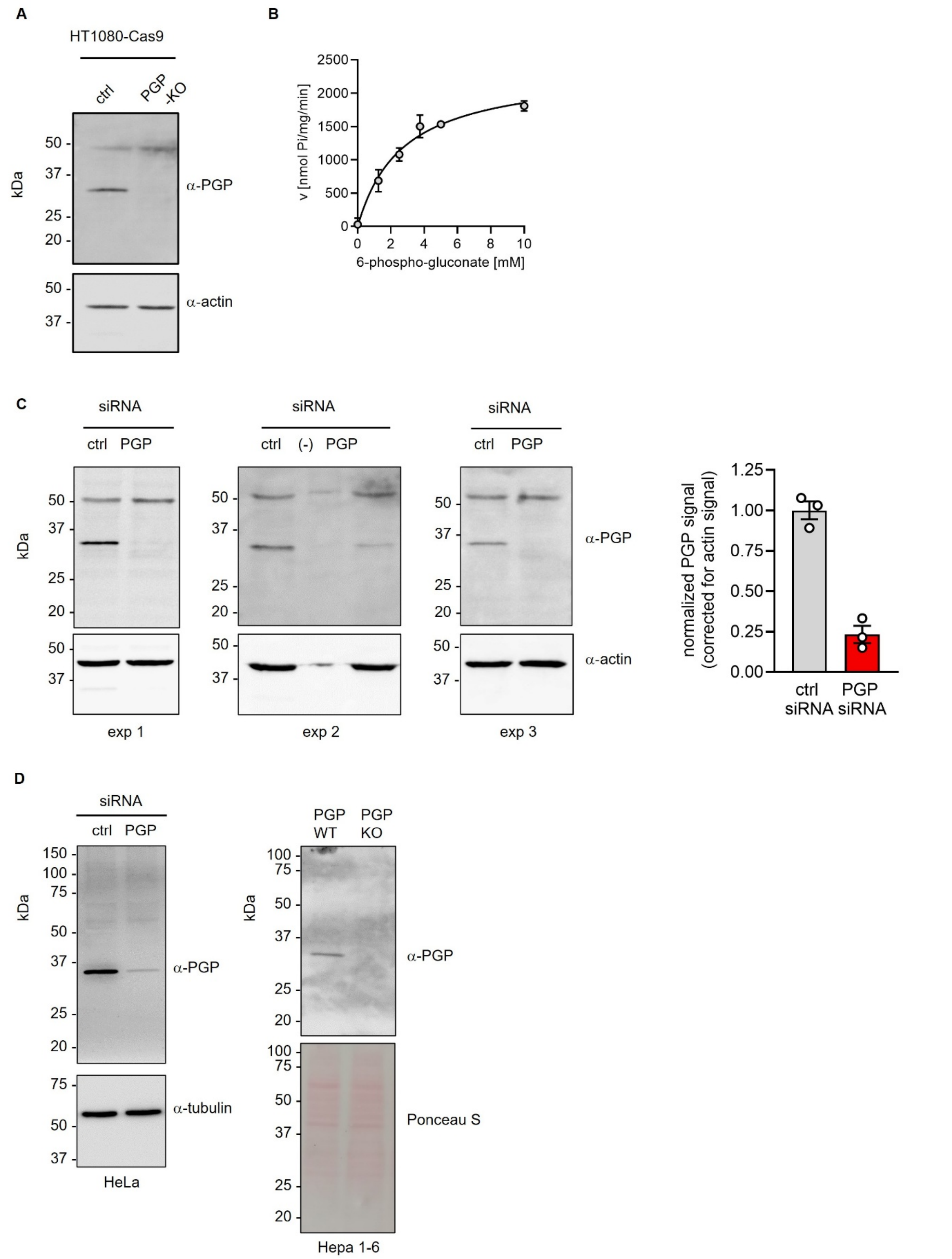

Fig. S1: Verification of PGP-knockout and PGP-knockdown in HT1080 cells and analysis of 6-phosphogluconate as a potential PGP substrate. (**A**) Western blot analysis of PGP expression in PGP-WT or PGP-KO HT1080 human fibrosarcoma cells generated by CRISPR/Cas9. The blot was first analyzed with α-PGP antibodies and then reprobed with α-actin antibodies as a loading control. (**B**) In vitro phosphatase activity assays of recombinant

purified murine PGP with 6-phosphogluconate as a substrate for the determination of the Michaelis-Menten constant (K_M) and maximal velocity (v_{max}). Phosphate release was measured using malachite green. Data are mean values ± S.E. of n=3 biologically independent experiments. Apparently missing error bars are hidden by the symbols. (**C**) Western blot analysis of PGP expression in HT1080 human fibrosarcoma cells transfected with control siRNA (ctrl) or PGP-siRNA. Blots were reprobed with α-actin antibodies as a loading control. The bar graph on the right shows the corresponding densitometric analysis of PGP expression levels (mean values ± S.E. of n=3 biologically independent experiments).

Fig. S2

A

			fold change: PGP-KO / PGP-WT					
lipid class	acyl carbons	acyl double bonds	0 h	1 h	2 h	4 h	6 h	ML210
	28	0	0.94	1.00	0.94	1.04	1.05	
	30	0	1.07	1.12	1.08	1.12	1.06	
		1	1.02	1.05	0.99	1.03	1.20	
	32	0	1.36	1.40	1.36	1.33	1.21	
		1	0.97	0.98	0.96	0.96	0.97	
		2	1.24	1.28	1.25	1.26	1.37	
	34	0	0.74	0.76	0.76	0.75	0.63	
		1	0.94	0.92	0.91	0.88	0.87	
		2	1.07	1.10	1.08	1.04	1.07	
		3	1.23	1.29	1.33	1.19	1.45	
		4	1.13	1.15	0.93	0.94	2.35	
	36	0	0.63	0.58	1.22	0.95	0.29	
		1	0.74	0.78	0.77	0.77	0.84	
		2	1.01	1.00	0.99	0.95	0.96	
		3	1.26	1.29	1.28	1.23	1.25	
	38	1	0.51	0.55	0.50	0.53	0.65	
		2	0.74	0.80	0.79	0.69	0.21	
		3	0.82	0.70	0.89	0.85	0.87	
		4	0.94	1.00	0.98	0.93	0.94	
PC		5	1.05	1.07	1.03	1.01	1.09	
		6	0.90	0.97	0.99	0.85	0.50	
	40	0	0.27	0.23	0.05	0.08	0.00	
		1	0.41	0.44	0.38	0.30	0.69	
		2	0.96	0.56	0.58	0.69	0.90	
		3	0.61	0.81	0.82	0.76	1.61	
		4	0.92	0.86	0.85	1.88	1.19	
		5	1.02	1.05	1.05	1.01	1.02	
		6	1.01	1.05	1.02	1.02	1.13	
		7	1.01	1.14	1.15	1.11	1.14	
		8	1.78	1.84	1.61	2.29	2.35	
	42	0	0.33	0.34	0.32	0.28	0.68	
		1	0.45	0.49	0.45	0.43	0.75	
		2	0.54	0.55	0.61	0.55	1.09	
		3	0.65	0.75	0.72	0.67	1.31	
		4	0.79	0.90	0.87	0.81	1.73	
		5	0.98	0.96	1.02	0.91	1.36	
		6	0.95	1.04	1.02	0.98	1.33	
		7	0.89	0.87	1.00	0.87	1.57	
		8	0.92	1.03	1.50	1.19	6.88	
	30	0	1.45	1.23	1.56	1.11	4.80	
	32	0	1.52	1.42	1.43	1.52	2.50	
		1	1.29	1.22	1.37	1.50	1.81	
	34	0	0.91	0.97	0.93	1.00	1.14	
		1	1.26	1.23	1.23	1.48	1.28	
		2	1.39	1.27	1.39	1.64	1.69	
		3	2.05	2.51	2.22	3.02		
	36	1	0.98	0.95	0.99	1.14	1.11	
		2	1.30	1.21	1.33	1.70	1.67	
		3	1.73	1.75	1.77	2.19	2.09	
		4	1.21	1.13	1.21	1.55	1.73	
	38	1	0.60	0.60	0.61	0.70	1.29	
PE		2	0.75	0.90	0.79	0.99	2.25	
		3	0.88	0.86	0.93	1.08	1.91	
		4	0.99	0.89	1.01	0.99	1.13	
		5	1.13	1.06	1.37	2.38	4.03	
		6	1.33	1.15	1.23	1.55	3.05	
	40	1	0.58	0.53	0.59	0.80	1.16	
		2	0.75	0.75	0.81	1.00	2.44	
		3	0.81	0.34	0.89	0.67	1.25	
		4	0.71	0.70	0.79	1.21	1.03	
		5	0.83	0.89	0.88	1.64	1.35	
	42	1	0.62	0.54	0.65	0.59	0.87	
		3	0.74	0.71	1.01	0.95		
		4	1.38	1.04	0.75	0.69	2.67	

C

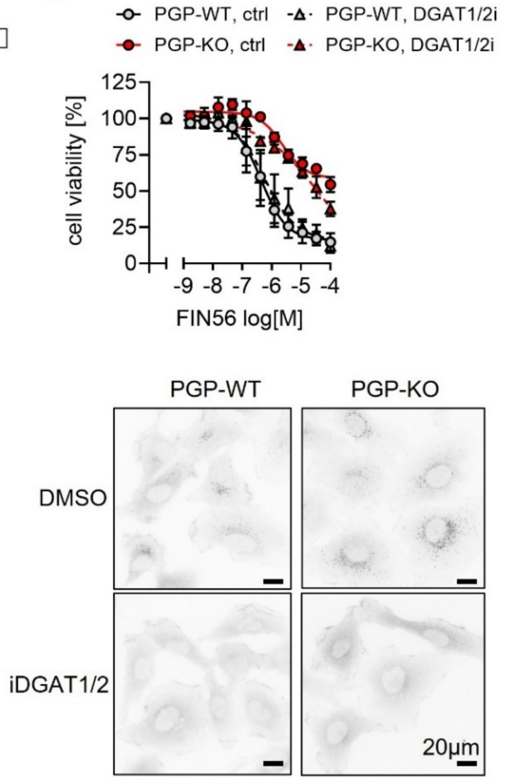

B

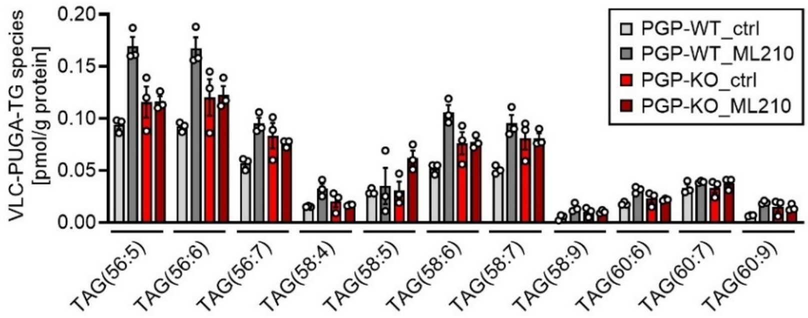

Fig. S2: Effect of PGP deletion on cellular lipids. **(A)** PGP-WT or PGP-KO HT1080 cells were treated with ML210 (0.5 µM) or the DMSO solvent control (0.1% v/v) for the indicated times, and cellular lipids were analyzed by mass spectrometry. Shown is the ML210-induced fold change of the indicated PC and PE species in PGP-KO versus PGP-WT in *n*=4 biologically

independent experiments. **(B)** Mass-spectrometric analysis of the abundance of very long-chain PUFA-TGs in PGP-WT or PGP-KO cells treated with ML210 (0.5 µM) or the DMSO solvent control (0.1% v/v) for 2.5 h. Data are mean values ± S.E. of *n*=3 biologically independent experiments. (**C**) Contribution of lipid droplet formation to ferroptosis sensitivity. Upper panel, HT1080 PGP-WT or PGP-KO cells were incubated for 16 h with the DMSO solvent control (0.1% v/v) or with inhibitors of DGAT1 (T863, 10 µM) and DGAT2 (PF-06424439, 5 µM) in the presence of serial dilutions of ML210. Cell viability was analyzed using resazurin. Apparently missing error bars are hidden by the symbols. Lower panel, Effect of inhibitors of DGAT1 (T863, 10 µM) and DGAT2 (PF-06424439, 5 µM) on lipid droplet formation in PGP-WT or PGP-KO HT1080 cells. Cells were fixed, lipid droplets were stained with an α-perilipin-3 antibody, and cells were imaged by epifluorescence microscopy. Images are representative of >20 imaged fields in *n*=3 biologically independent experiments.

Fig. S3

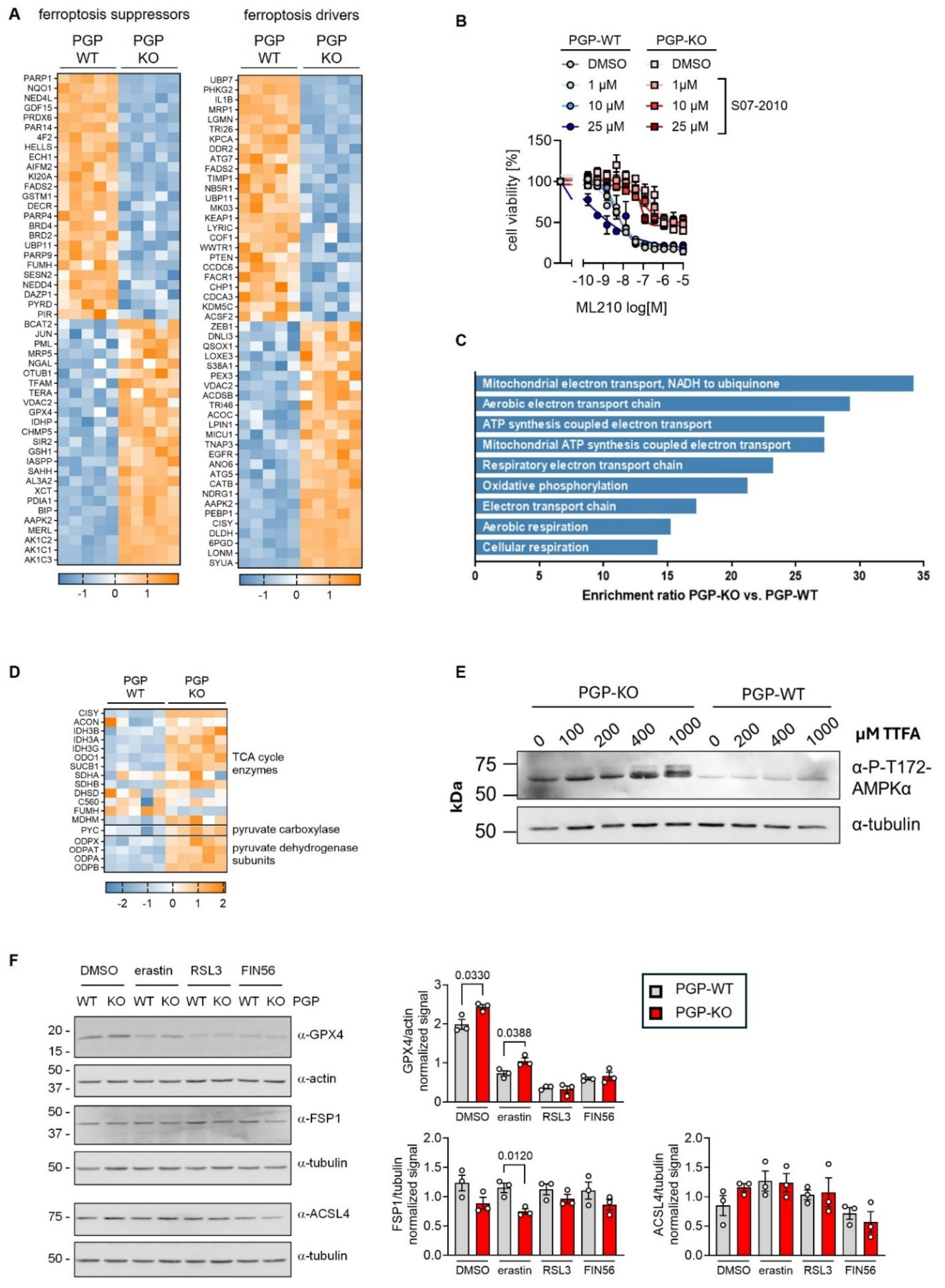

Fig. S3: Effect of PGP loss on ferroptosis regulators. (**A**) Whole-cell proteomes of PGP-WT and PGP-KO HT1080 cells were matched against the ferroptosis database (see Materials and Methods for details), and ferroptosis drivers or -suppressors were extracted. A heatmap representation with the z-scores of the top 25 up- or downregulated proteins per category is

shown. All data are from mean values in *n*=5 biologically independent experiments. (**B**) HT1080 PGP-WT and PGP-KO cells were incubated with the indicated concentrations of the pan-AKR1C inhibitor S07-2010 or the DMSO control, and ferroptosis was induced with serial dilutions of the GPX4 inhibitor ML210. Cell viability was analyzed using resazurin. All data are mean values ± S.E. of *n*=3 biologically independent experiments. Apparently missing error bars are hidden by the symbols. (**C**) Biological pathway enrichment analysis. The top 100 upregulated proteins in HT1080 PGP-KO versus PGP-WT cells were subjected to an over-representation analysis against the total proteome of HT1080 PGP-WT cells. All data are based on *n*=5 biologically independent experiments. (**D**) Heatmap representation of TCA cycle enzyme expression in the proteomes of PGP-WT and PGP-KO HT1080 cells. Z-scores are shown. Data are mean values from *n*=5 biologically independent experiments. (**E**) Analysis of Thr172-P-AMPK and AMPK levels in PGP-WT or PGP-KO cells treated for 30 min with the indicated concentrations of the complex II inhibitor thenoyltrifluoroacetone/TTFA. Tubulin was used as a loading control on the same membrane. (**F**) Left panels, representative Western blots of GPX4, FSP1 and ACSL4 in lysates of PGP-WT or PGP-KO HT1080 cells treated for 16 h with 100 nM liproxstatin and the solvent control (0.1% v/v DMSO) or the indicated ferroptosis inducers (1 µM erastin, 1 µM RSL3, 10 µM FIN56). Blots were reprobed with α-actin or α-tubulin antibodies as loading controls. Right panel, densitometric analysis of Western blots. Data are mean values ± S.E. of *n*=3 biologically independent experiments. The blots of the other two experiments are shown in table S3. Statistical analysis in **F**: unpaired, two-sided *t*-tests, *p*-values are indicated.

Fig. S4

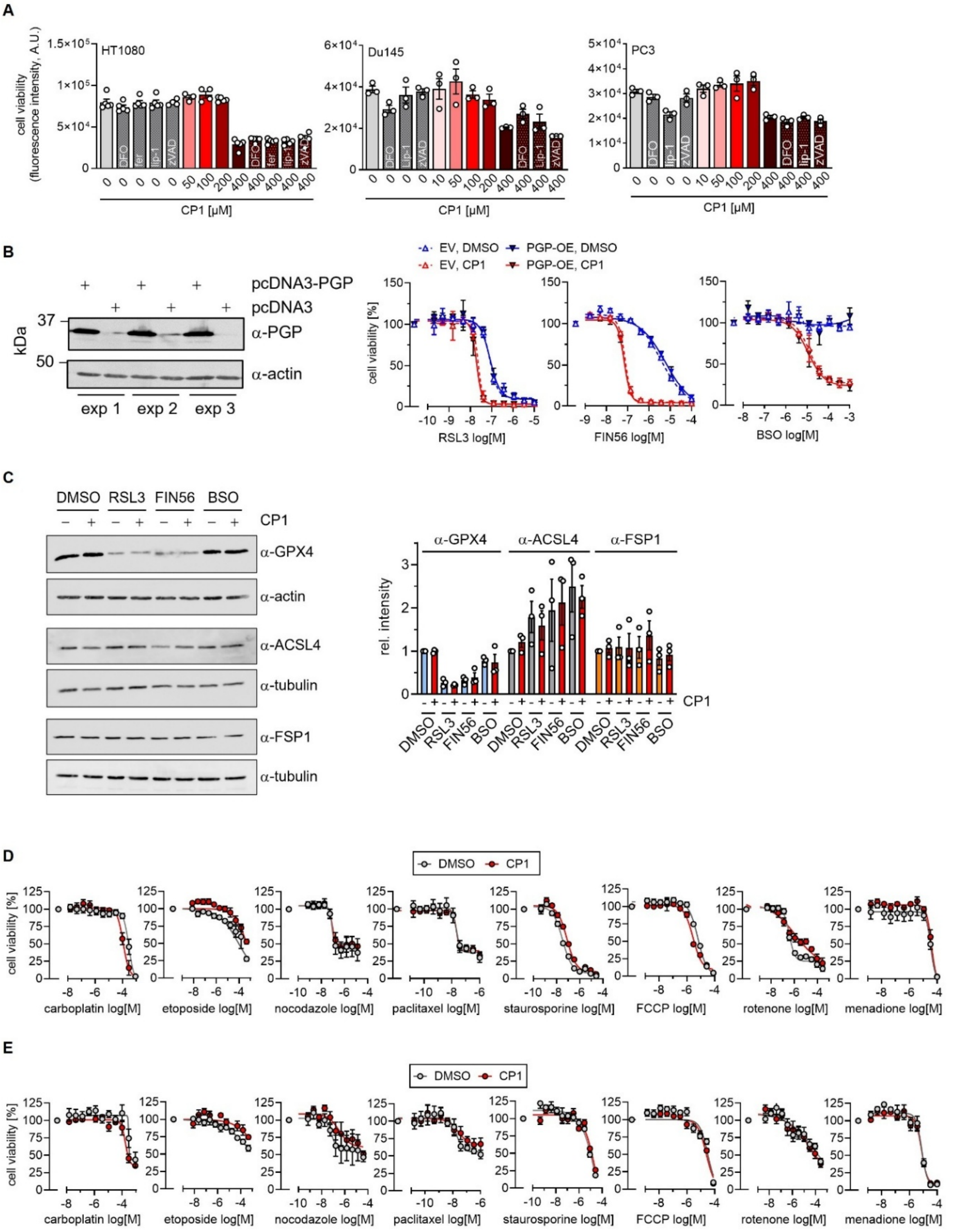

Fig. S4: Characterization of CP1. (**A**) Effect of increasing CP1 concentrations on the viability of HT1080, Du145 or PC3 cells. Cells were incubated for 16 h with the indicated concentrations of CP1, ferroptosis or apoptosis inhibitors, and cell viability was analyzed using AquaBluer. DMSO solvent control: 0.4% v/v under all conditions. DFO, deferoxamine (200 µM); fer, ferrostatin (200 nM); lip-1, liproxstatin-1 (200 nM); zVAD, zVAD-FMK (100 µM). (**B**) Left panel, Western blot analysis of HT1080 cells transiently transfected with either the empty control vector (EV) or with PGP (PGP overexpression, PGP-OE). Exp, experiment.

Right panels, cells were incubated for 16 h with serial dilutions of the indicated ferroptosis inducers in the absence (0.1% v/v DMSO) or presence of CP1 (100 μM). Cell viability was analyzed using Aquabluer. Data are mean values ± S.E. of *n*=3 biologically independent experiments. (**C**) Left panels, representative Western blots of GPX4, ACSL4, or FSP1 in parental HT1080 cells. Cells were cultured in the presence of liproxstatin-1 (100 nM) and incubated for 16 h with the solvent control (0.1% v/v DMSO) or the indicated ferroptosis inducers (1 μM RSL3, 10 μM FIN56, 100 μM BSO) ± solvent control (0.1% v/v DMSO) or CP1 (100 μM). Blots were reprobed with α-actin or α-tubulin antibodies as loading controls. Right panels, densitometric analysis of Western blots. Data are mean values ± S.E. of *n*=3 biologically independent experiments. (**D**) HT1080 or (**E**) PC3 cells were incubated for 16 h with the indicated panel of cytotoxic drugs, together with DMSO (0.1% v/v) or CP1 (100 μM), and cell viability was analyzed with Aquabluer. FCCP, carbonyl cyanide 4-(trifluoromethoxy) phenyl-hydrazone. All data are mean values ± S.E. of *n*≥3 biologically independent experiments. Apparently missing error bars are hidden by the symbols.

Fig. S5

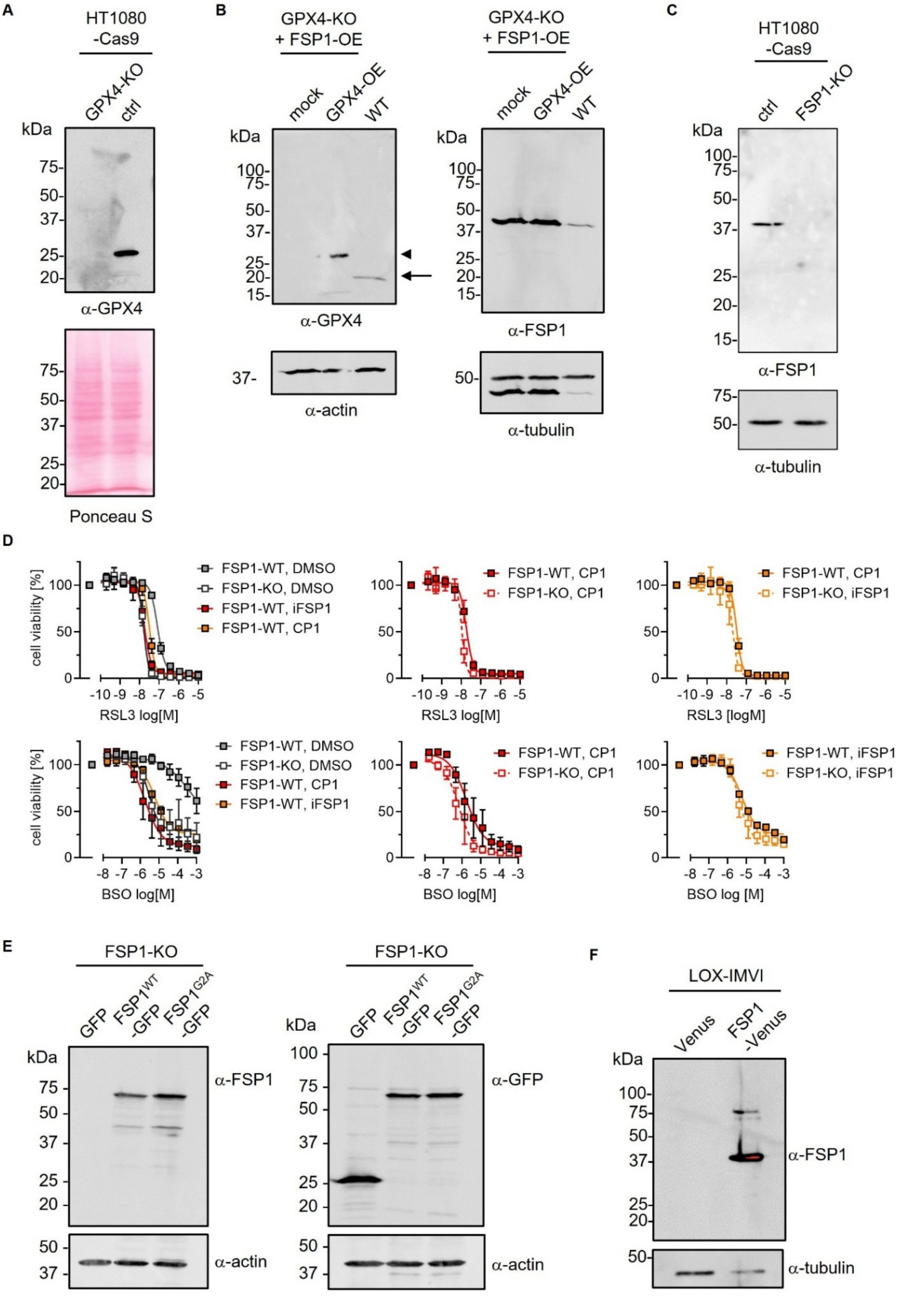
A
HT1080 -Cas9
GPX4-KO
ctrl
kDa
75-
50-
37-
25-
20-
α-GPX4
Ponceau S
B
GPX4-KO + FSP1-OE
mock
GPX4-OE
WT
100-
15-
α-GPX4
α-actin
α-FSP1
α-tubulin
C
FSP1-KO
D
cell viability [%]
FSP1-WT, DMSO
FSP1-KO, DMSO
FSP1-WT, iFSP1
FSP1-WT, CP1
FSP1-KO, CP1
FSP1-KO, iFSP1
RSL3 log[M]
RSL3 [logM]
BSO log[M]
BSO log [M]
E
FSP1-KO
GFP
FSP1WT -GFP
FSP1G2A -GFP
α-GFP
F
LOX-IMVI
Venus
FSP1 -Venus

Fig. S5. Employed cell lines and effect of CP1 on FSP1-dependent ferroptosis. (**A-C; E, F**), Western blot analyses. Note that the reconstituted GPX4 (in GPX4-KO/FSP1-OE/GPX4-OE cells) in **fig. S5B** is Flag-Streptavidin-HA (FSH)-tagged (arrowhead), leading to the mobility shift compared to parental HT1080 expressing endogenous GPX4 (arrow). (**D**) Comparison of CP1 and iFSP1 on RSL3 or BSO-induced ferroptosis in HT1080 FSP1-WT or FSP1-KO cells. For better visibility, FSP1-WT or FSP1-KO cells treated with CP1 (100 μM) or iFSP1 (10 μM) are shown separately in the middle or right panel, respectively. Note that the curves of inhibitor-treated FSP1-WT cells are shown for comparison with the inhibitor-treated FSP1-KO cells and are identical to the respective curves shown in the left panel. Data are means ± S.E. of $n \geq 3$ biologically independent experiments. Cell viability was assessed with Aquabluer after 16 h incubation with the indicated compounds. Apparently missing error bars are hidden by the symbols.

Fig. S6

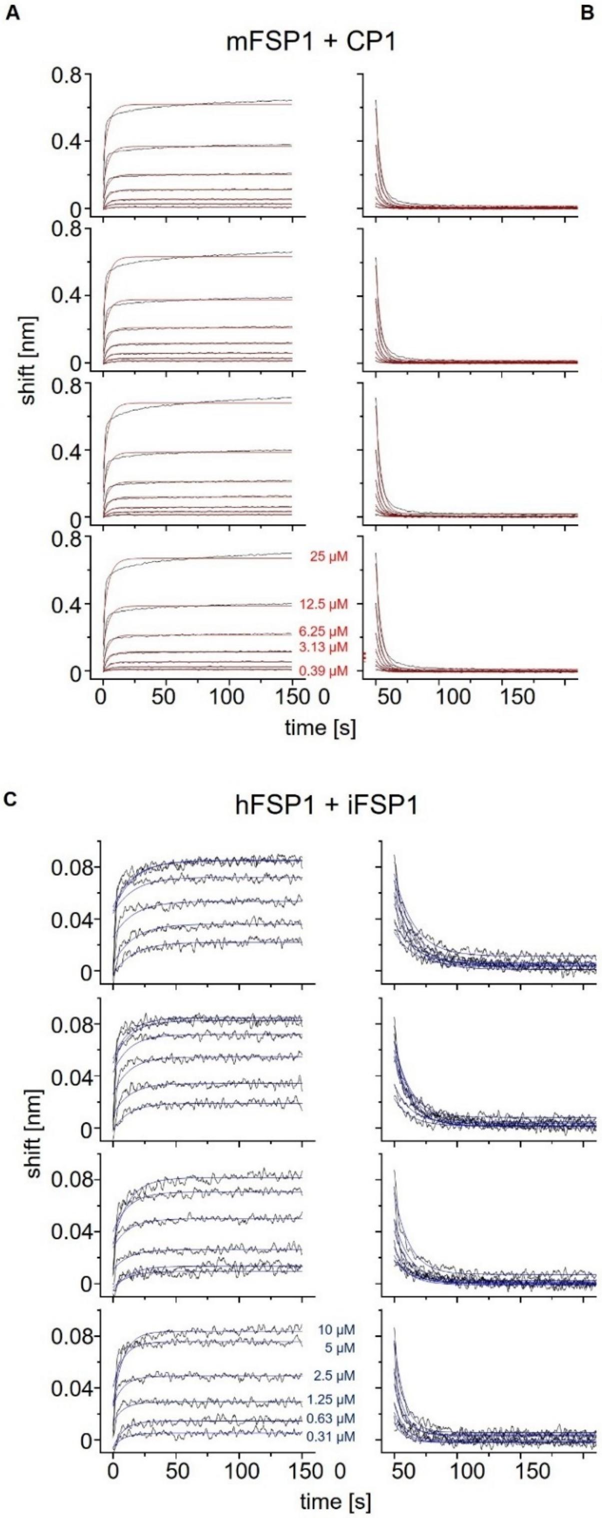

Fig. S6. Biolayer interferometry measurements of the interaction of FSP1 with CP1 or iFSP1. Interaction of CP1 with (**A**) murine FSP1 (mFSP1) or (**B**) human FSP1 (hFSP1), or (**C**), of iFSP1 with hFSP1. Recombinant, purified, biotinylated Avi-tagged FSP1 was used. Left panels, association sensorgrams (black) overlayed with the global 1:1 association binding model. Right panels, dissociation sensorgrams (black) overlayed with the global 1:1 dissociation binding model. Red, CP1; blue, iFSP1. Data are from *n*=4 technically independent measurements.

Fig. S7

A

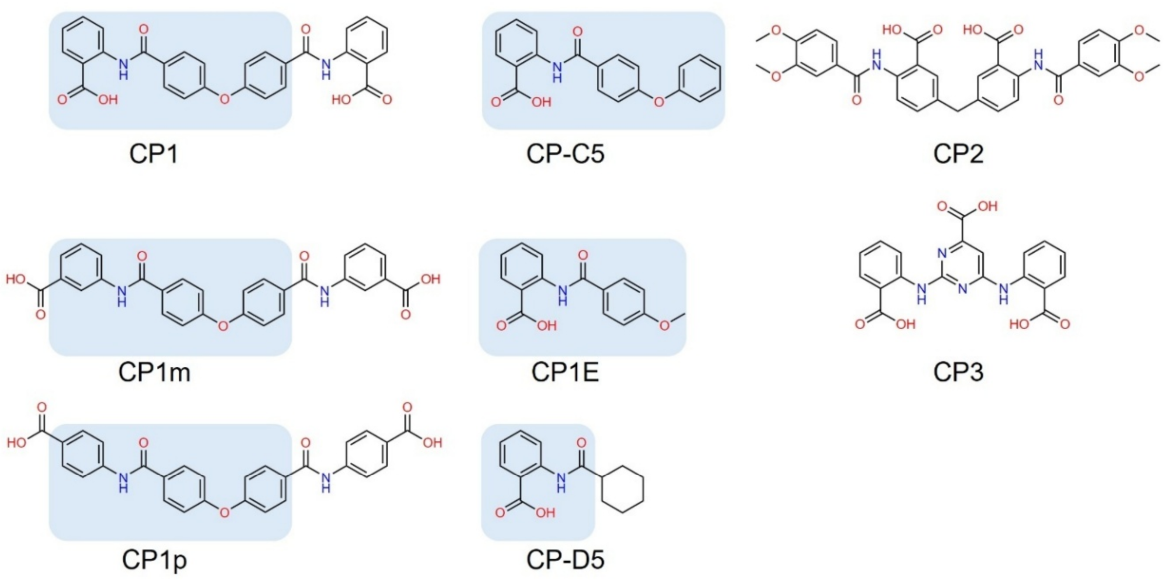

B

CP	Inhibition [%]			IC_{50} [µM]			Selectivity
	mFSP1	hFSP1	mPGP*	mFSP1	hFSP1	mPGP	mPGP/mFSP1
CP1	96.9 ± 1.2	76.3 ± 5.8	97.4 ± 2.2*	2.2 ± 0.3	31.1 ± 4.5	0.4 ± 0.1*	5.5
CP1m	15.8 ± 13.2	8.5 ± 17.0	62.9 ± 3.0*	n.d.	n.d.	27.8 ± 6.3*	
CP1p	15.3 ± 16.8	5.1 ±17.6	55.4 ± 2.4	n.d.	n.d.	46.3 ± 8.9	
CP-C5	75.0 ± 5.6	45.3 ± 7.5	52.7 ± 1.9	11.0 ± 2.3	84.1 ± 14.5	43.5 ± 2.6	0.25
CP1E	34.7 ± 1.5	21.4 ± 8.0	38.8 ± 3.0.*	n.d.	n.d.	n.d.	
CP-D5	57.0 ± 1.5	25.0 ± 15.8	0 ± 4.9	70.3 ± 26.4	n.d.	n.d.	
CP2	76.3 ± 7.9	4.9 ± 8.1	90.3 ± 0.6*	38.7 ± 5.1	n.d.	1.6 ± 0.3*	24.2
CP3	5.6 ± 10.7	0 ± 3.2	97.5 ± 2.4*	> 100	n.d.	0.8 ± 0.1*	> 100

C

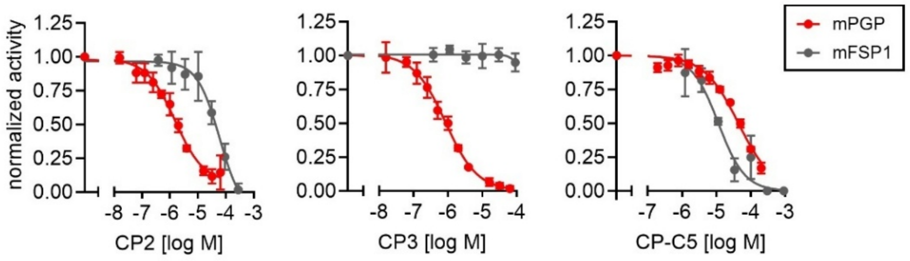

Fig. S7: Structure-activity relationship studies. (**A**) 2D structures of CP1, CP1 analogs, CP2 and CP3. Common structural elements are shaded in blue. (**B**) The effects of the indicated compounds on FSP1 oxidoreductase activity were assessed in NADH consumption assays, using recombinant, purified murine or human FSP1 (mFSP1, hFSP1) and resazurin as an

electron acceptor. Compounds were initially tested at a concentration of 100 µM, and the percentage of FSP1 or PGP inhibition under these conditions is indicated. IC_{50} values were determined if the compound inhibited FSP1 activity by more than 50% compared to the DMSO solvent control. All results are mean values ± S.D. of *n*=3 biologically independent experiments; n.d., not determined. * PGP data are taken from (*35*). (**C**) Determination of CP2, CP3 or CP-C5 IC_{50} values for murine PGP (mPGP) or mFSP1, using recombinant, purified enzymes. FSP1: NADH consumption assays with resazurine as an electron acceptor; PGP: phosphatase assays using phosphoglycolate as a substrate and malachite green to detect free inorganic phosphate. FSP1 oxidoreductase or PGP phosphatase activities in the presence of CP2, CP3 or CP-C5 were normalized to the respective enzyme activities measured in the presence of the solvent control DMSO (0.1% v/v). Data are mean values ± S.D. of *n*=3-4 biologically independent experiments.

Fig. S8

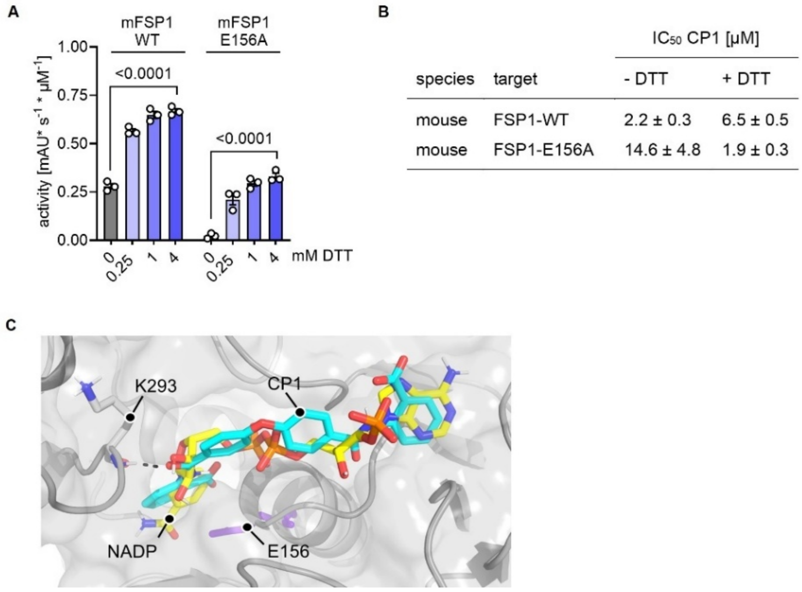

Fig. S8: Characterization of FSP1-E156A and docking of CP1 to FSP1. (**A**) Analysis of the oxidoreductase activity of purified mFSP1-E156A in comparison with murine FSP1 in the presence of increasing DTT concentrations. Statistical analysis was performed with an unpaired, two-tailed *t*-test; *p*-values are indicated. (**B**) Determination of the CP1 IC_{50} values of murine FSP1 and its E156A point mutant in the absence or presence of 4 mM DTT. (**A**, **B**): DMSO was used as a CP1 solvent control and was kept constant in each experiment. Results were normalized to the protein input (3 µM). Data are mean values ± S.E. of *n*=3 biologically independent experiments. (**C**) Docking of CP1 to FSP1. The top-scoring docking pose of CP1 (cyan) is superimposed with the binding pose of NADP (yellow) from the hFSP1-NADP complex (PDB 8JSC). Glu156 is shown in purple. The displayed CP1 binding pose is representative of the five best-ranked poses.

Fig. S9

A

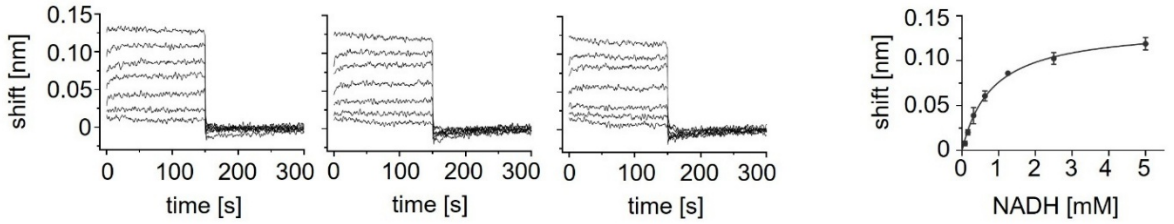

B

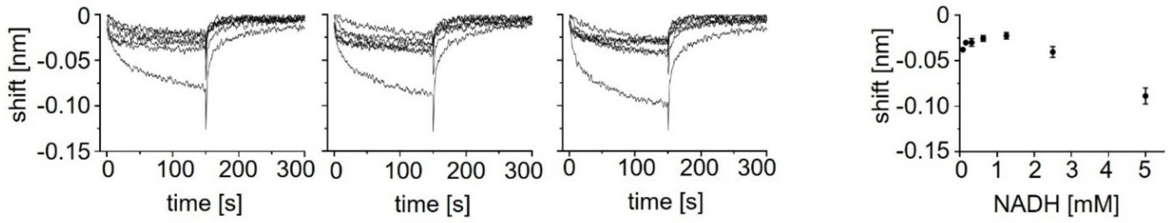

C

K_D NADH [μM]	R max [nm]	R^2
819.6 ± 73.2	0.1385 ± 0.0042	0.984

Fig. S9: Biolayer interferometry measurements of the FSP1/NADH interaction in the absence or presence of CP1. (**A**) Concentration-response analysis of NADH binding to mFSP1 and (**B**) of NADH binding to mFSP1 in the presence of 50 μM CP1. Left panels, the obtained shifts are plotted against the NADH concentration. Shown are the sensorgrams after alignment and double referencing. Right panels, fit to the Langmuir model. Data are mean values ± S.D. of *n*=3 technically independent measurements. (**C**) Obtained binding parameters for the mFSP1/NADH interaction.

Fig. S10

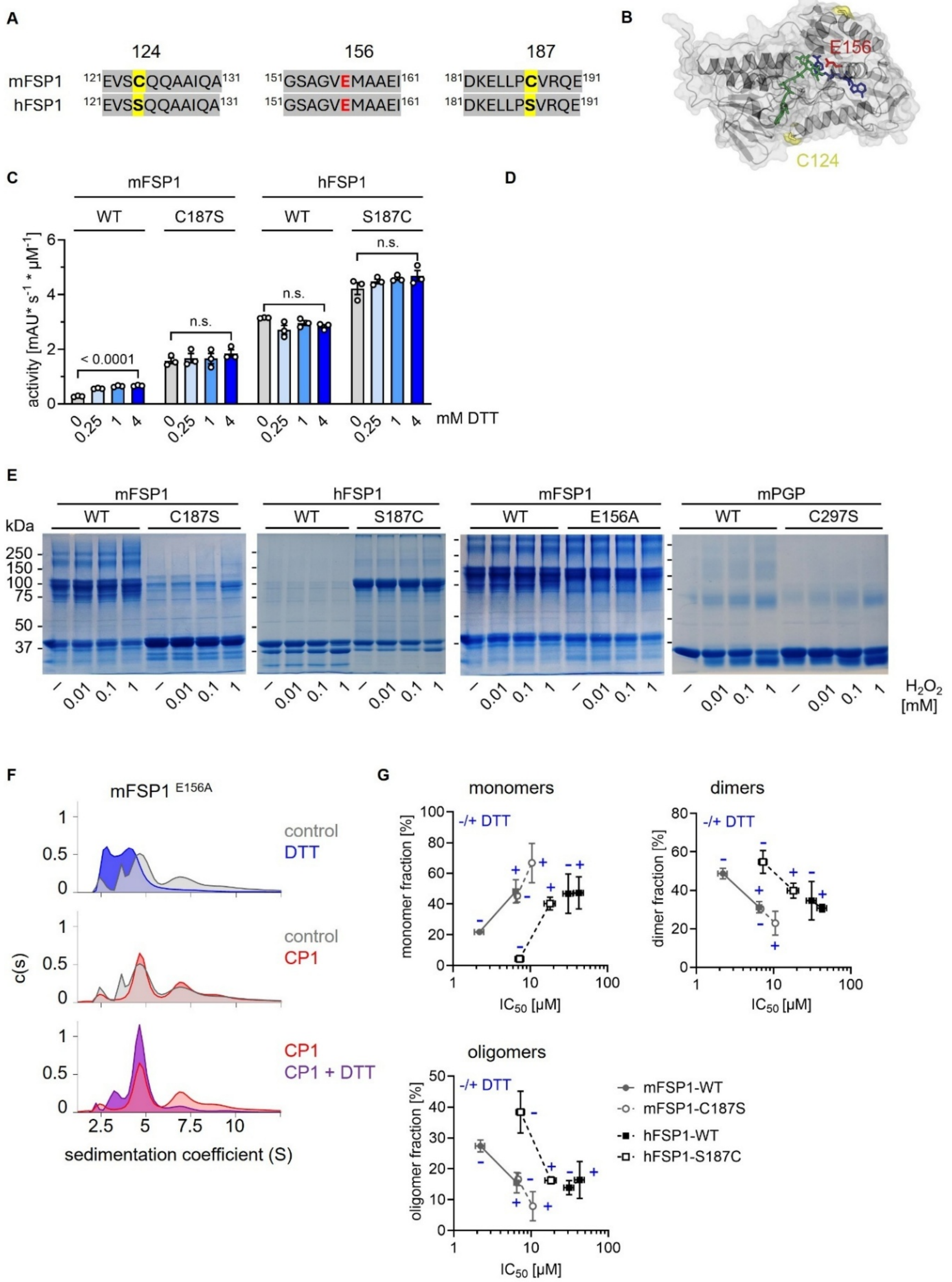

Fig. S10: Effects of CP1 on FSP1. (**A**) Sequence alignment of murine FSP1 (UniProt-ID Q8BUE4) and human FSP1 (UniProt-ID Q9BRQ8) in the vicinity of the conserved Glu156 residue of the proton transfer chain and the surface-exposed Cys124/Cys187 residues of murine FSP1. (**B**) Cartoon and semi-transparent surface representation of the AlphaFold model structure of murine FSP1. 6-Hydroxy-FAD (green, stick representation) and NADPH (blue,

stick representation) were obtained by superimposing the AlphaFold model with the human FSP1 X-ray crystal structure (PDB code 8YOQ). (**C**) Oxidoreductase activity of the indicated, purified enzymes in the presence of increasing DTT concentrations. Statistical analysis: unpaired, two-tailed *t*-test, *p*-values are indicated. (**D**) Determination of the CP1 IC_{50} of the indicated, purified enzymes in the absence or presence of 4 mM DTT. (**C, D**): DMSO was used as a CP1 solvent control and was kept constant in each experiment. Results were normalized to the protein input (final mFSP-WT concentration, 3 µM; all other FSP1 proteins, 0.3 µM). Data are mean values ± S.D. of n=3 biologically independent experiments. (**E**) Analysis of the mFSP1 and hFSP1 oligomerization profile with non-reducing SDS-PAGE. Proteins (10 µg) were pre-incubated with H_2O_2 as indicated. Coomassie Blue-stained gels are shown. (**F**) Analytical ultracentrifugation (AUC) analysis. mFSP1-E156A (10 µM protein per condition) was incubated with the DMSO solvent control (0.2% v/v) or DTT (5 mM) ± CP1 (100 µM). Mean values of n=3 biologically independent experiments are shown. FSP1 dimers correspond to peak sedimentation coefficients around 4.5 S. See fig. S11 for histograms of all AUC experiments. (**G**) Plotting of FSP1 monomer, dimer and oligomer fractions estimated from AUC profiles (see fig. 10C) against the CP1 IC_{50} values of the respective proteins (see fig. S10D). Note that FSP1 proteins with a higher proportion of dimers are more sensitive to CP1 inhibition and DTT treatment than FSP1 proteins with a high proportion of monomers. Mean values ± S.D. of n=2-3 independent experiments are shown.

Fig. S11

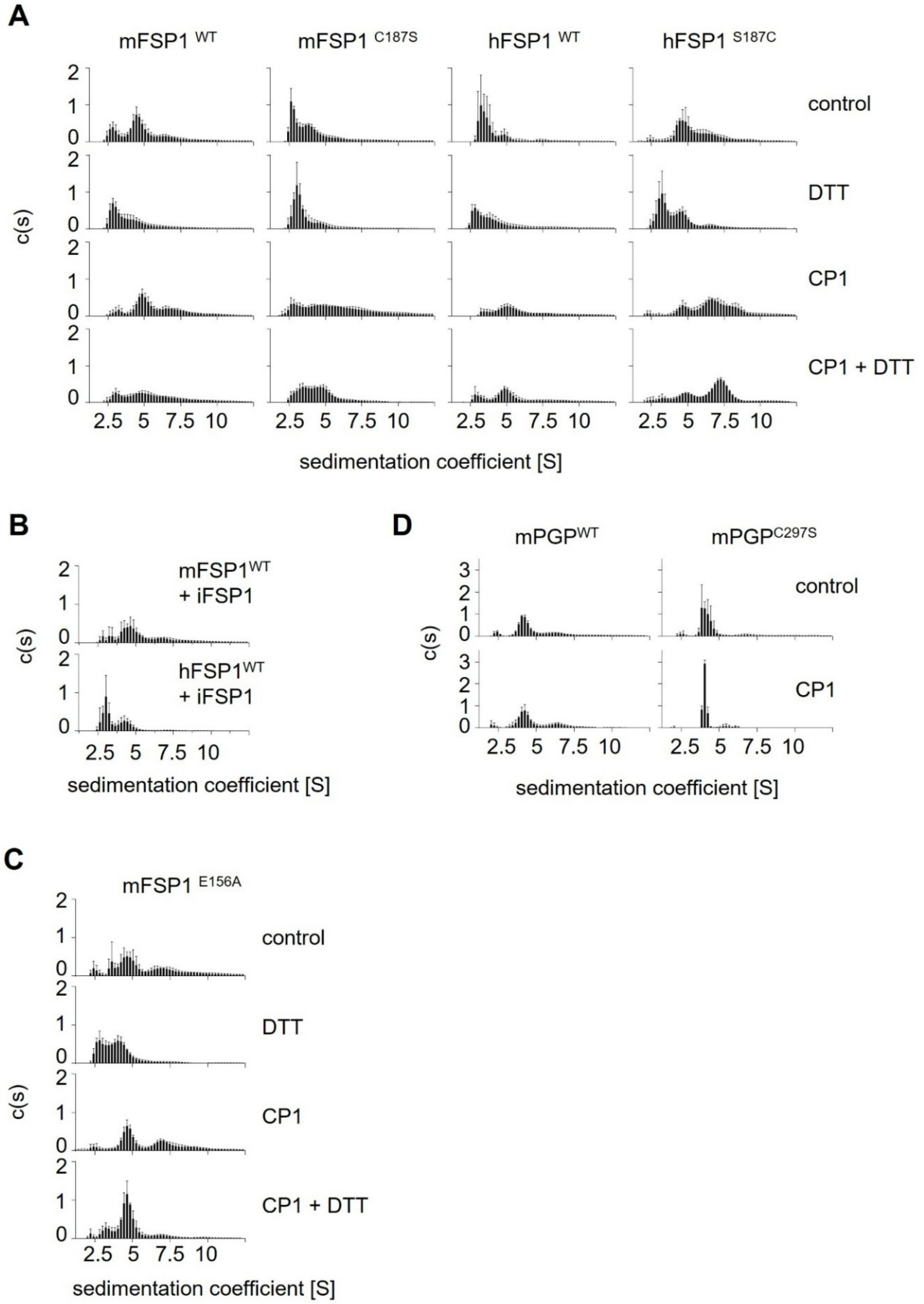

Fig. S11: Sedimentation velocity analytical ultracentrifugation (AUC) analysis of FSP1 and PGP. Shown are continuous sedimentation coefficient distribution analyses of the indicated proteins. (**A-C**) FSP1 proteins (10 µM protein each) were incubated with the DMSO

solvent control (0.2% v/v), DTT (5 mM), CP1 (100 µM) or iFSP1 (100 µM). The histograms show the mean values ± S.D. of n=3 biologically independent experiments, except for hFSP1^{S187C} + DTT and hFSP1^{S187C} + CP1 + DTT (n=2 biologically independent experiments). (**D**) mPGP-WT or mPGP-C297S (9 µM protein each) were incubated with the DMSO solvent control (0.2% v/v) or with 100 µM CP1. The histograms show the mean values ± S.D. of n=3 biologically independent experiments.

Table S1: Mass spectrometric analysis of water-soluble metabolites.

Table S1		PGP-WT	PGP-KO	PGP-WT	PGP-KO	
		mean	mean	SE	SE	p-value
PPP, glycolysis	Phosphoerythronate	0.00	48.43	0.00	21.39	**0.0002**
	Phosphogluconate	220.60	17439.72	242.75	831.35	**<0.0001**
	Gluconate	903.14	38112.95	71.58	7550.27	**<0.0001**
	Phosphogluconolactone	0.00	17.24	0.00	17.24	**0.0343**
	Erythrose-4-P	87.14	52.98	30.44	40.24	0.1282
	Erythronate	154.40	205.63	65.97	234.63	0.6179
	Pentose-P	24.84	74.28	8.47	14.13	**<0.0001**
	Seduheptulose-7-P	21.62	29.29	14.44	17.97	0.4337
	Glyceral/-one-P/P-lactate	330.13	4673.57	178.93	2098.81	**0.0005**
	Glycerol-3-P	146.41	69.66	19.38	12.69	**<0.0001**
	Pyruvate	12455.82	4444.27	7394.08	2002.51	**0.0283**
	Lactate	58037.10	32894.50	34194.87	21363.69	0.1576
NAD(P)H,glutathione	NADPH	0.00	1.86	0.00	1.59	**0.0169**
	NADP	17.51	21.85	8.28	8.84	0.3999
	NADPH/NADP+	0.00	0.13	0.00	0.13	**0.0356**
	Cystine	11.91	22.85	15.05	9.32	0.1609
	GSH	8462.48	14524.19	3161.35	939.16	**0.0011**
	GSSG	1213.09	912.29	879.57	105.14	0.4250
	GSH/GSSG	10.29	16.10	5.75	2.23	**0.0435**
transsulfuration	Methionine	11925.25	11686.62	9293.28	6798.01	0.9605
	SAM	234.64	428.47	141.02	253.94	0.1332
	SAH	15.34	14.10	9.60	4.09	0.7773
	SAM/SAH	0.11	0.05	0.10	0.03	0.1759
	Cystathionine	392.27	805.09	57.09	75.17	**<0.0001**
	Homocysteine	6.82	7.73	7.77	8.49	0.8507
glutamylated amino acids	GluAla	254.36	165.09	239.59	136.76	0.4464
	GluArg	153272.10	121617.45	169444.91	133356.80	0.7266
	GluAsn	88.77	84.28	37.02	23.15	0.8062
	GluAsp	477.57	420.17	274.94	194.94	0.6854
	GluCys	6.62	20.01	4.61	8.78	**0.0079**
	GluGln	136.73	96.67	113.37	65.34	0.4706
	GluGlu	468.96	358.74	442.13	315.03	0.6297
	GluGly	165.38	143.02	138.93	111.12	0.7645
	GluHis	112.23	60.57	116.88	40.71	0.3306
	GluIle&GluLeu	160.50	109.57	159.96	97.95	0.5210
	GluLys	36.49	38.77	27.31	23.72	0.8803
	GluMet	1.83	2.00	2.14	2.28	0.8987
	GluPhe	17.63	18.79	15.18	16.37	0.9010
	GluPro	192.70	418.86	377.22	382.50	0.3267
	GluSer	150.69	127.64	82.19	51.94	0.5742
	GluThr	60.79	51.36	31.67	23.47	0.5711
	GluTrp	0.13	0.10	0.31	0.25	0.8739
	GluTyr	16.02	15.00	3.38	1.73	0.5234
	GluVal	134.55	96.48	115.66	76.79	0.5170
acetylated amino acids	N-Ac-Asn	4.49	3.23	5.01	3.54	0.6244
	N-Ac-Asp	5453.93	5502.35	2141.19	1751.74	0.9666
	N-Ac-Cys	45.36	87.31	15.45	11.98	**0.0004**
	N-Ac-Glu	322.49	249.34	166.38	29.40	0.3139
	N-Ac-Gly	9.41	8.22	9.15	8.23	0.8172
	N-Ac-Ile/N-Ac-Leu	9.45	3.49	11.38	3.75	0.2510
	N-Ac-Lys	608.13	445.35	512.78	304.78	0.5190
	N-Ac-Met	388.73	389.62	24.76	124.12	0.9866
	N-Ac-Phe	6.70	4.04	3.06	2.89	0.1537
	N-Ac-Orn	178.13	123.13	212.15	80.44	0.5659
amino acids	Alanine	2353.94	2097.59	505.13	299.67	0.3102
	Arginine	28498.22	25512.44	21395.76	15838.72	0.7891
	Asparagine	625.01	831.25	159.47	178.67	0.0611
	Aspartate	1597.14	1844.54	196.51	264.90	0.0960
	Cysteine	11.80	17.46	16.68	19.26	0.5984
	Glutamate	52061.51	56475.73	11740.15	15148.20	0.5851
	Glutamine	57343.01	60229.60	28956.89	33031.06	0.8753
	Glycine	1253.33	1641.89	748.11	1009.29	0.4662
	Proline	5692.17	8496.46	4173.68	5831.32	0.3607
	Serine	1628.87	1378.80	461.07	316.65	0.2991
	Tyrosine	8808.83	10385.62	4781.36	4376.87	0.5645
	Histidine	3123.54	9864.09	2566.04	6986.10	0.0508
	Isoleucine	44971.96	44300.31	15170.49	6406.82	0.9224
	Leucine	31014.56	35910.68	17315.65	15666.06	0.6187
	Lysine	7948.49	8380.64	3890.18	2907.93	0.8319
	Methionine	11925.25	11686.62	9293.28	6798.01	0.9605
	Methioninesulfoxide	192.94	279.71	34.96	57.73	**0.0103**
	Phenylalanine	26081.45	29020.83	13363.75	10841.25	0.6845
	Threonine	6855.69	7601.26	1373.33	474.29	0.2373
	Tryptophan	5398.47	6627.34	998.76	509.74	**0.0229**
	Valine	4974.95	5914.35	257.93	1260.36	0.1040
	2-Methylbutyrylglycine	56.59	41.98	34.16	13.01	0.3505

	ctrl siRNA	PGP siRNA	ctrl siRNA	PGP siRNA	
	mean	mean	SE	SE	p-value
Phosphoerythronate	0.14	4.67	0.34	4.47	**0.0326**
Phosphogluconate	178.54	1526.08	73.49	1046.53	**0.0104**
Gluconate	1107.91	2646.39	399.11	1961.19	0.0891
Phosphogluconolactone	0.03	0.00	0.08	0.00	0.3409
Erythrose-4-P	160.67	125.13	114.55	87.07	0.5586
Erythronate	17.42	14.23	15.14	13.53	0.7085
Pentose-P	34.75	72.28	12.98	35.58	**0.0357**
Seduheptulose-7-P	48.92	34.68	29.93	10.76	0.2983
Glyceral/-one-P/P-lactate	919.26	6990.13	578.32	4439.24	**0.0077**
Glycerol-3-P	263.22	121.25	93.04	17.21	**0.0043**
Pyruvate	23714.50	16062.48	7218.52	2155.56	**0.0321**
Lactate	118536.01	93860.36	75925.57	59799.01	0.5457
NADPH	2.25	3.96	5.51	5.73	0.6093
NADP	64.40	57.06	6.01	6.25	0.0652
NADPH/NADP+	0.04	0.07	0.09	0.12	0.5480
Cystine	2.76	0.67	4.74	0.81	0.3120
GSH	23817.94	25229.22	7241.02	6438.27	0.7287
GSSG	1191.86	1069.38	382.85	300.76	0.5515
GSH/GSSG	20.36	23.92	2.94	3.00	0.0648
Methionine	6793.93	8050.51	1362.48	819.52	0.0816
SAM	869.87	964.76	211.72	208.83	0.4526
SAH	52.53	51.73	24.45	20.75	0.9524
SAM/SAH	0.06	0.05	0.02	0.01	0.5802
Cystathionine	734.51	907.16	250.85	290.05	0.2959
Homocysteine	0.20	0.34	0.30	0.23	0.3771

Results are from PGP-WT versus PGP-KO HT1080 cells or PGP-siRNA versus control siRNA HT1080 cells, respectively. Note that glyceral phosphate, glycerone phosphate and phospholactate are isomeric and cannot be unequivocally identified with the employed method. All metabolite levels are given as peak areas normalized to total metabolites. All data are means ± SE of *n*=6 biologically independent experiments. Statistical significance was assessed with unpaired, two-tailed *t*-tests.

Table S2. Effect of CP1 on FSP1 enzyme kinetics.

(**A**)

	CP1 [µM]	0	1	3	6
mFSP1 WT	v_{max} [mAU· s^{-1}·μM^{-1}]	0.613 ± 0.051	0.487 ± 0.035	0.340 ± 0.017	0.173 ± 0.016
	K_M [µM]	39.3 ± 10.9	29.3 ± 8.0	25.0 ± 5.2	6.90 ± 4.99

	CP1 [µM]	0	1	3	6
mFSP1 C187S	v_{max} [mAU· s^{-1}·μM^{-1}]	1.76 ± 0.13	1.57 ± 0.09	0.751 ± 0.037	0.341 ± 0.019
	K_M [µM]	73.0 ± 14.9	58.9 ± 9.7	14.0 ± 3.7	0.571 ± 1.81

	CP1 [µM]	0	5	30	60
hFSP1 WT	v_{max} [mAU· s^{-1}·μM^{-1}]	4.70 ± 0.14	2.83 ± 0.12	1.62 ± 0.10	1.29 ± 0.11
	K_M [µM]	50.0 ± 4.7	41.8 ± 6.2	19.4 ± 5.5	8.64 ± 4.81

	CP1 [µM]	0	1	3	6
hFSP1 S187C	v_{max} [mAU· s^{-1}·μM^{-1}]	5.10 ± 0.26	3.22 ± 0.26	2.40 ± 0.11	1.79 ± 0.09
	K_M [µM]	34.4 ± 6.2	31.0 ± 9.5	16.4 ± 3.5	5.20 ± 1.78

(**B**)

	CP1 [µM]	0	1	3	6
mFSP1 WT	v_{max} [mAU· s^{-1}·μM^{-1}]	0.683 ± 0.092	0.668 ± 0.085	0.739 ± 0.160	0.633 ± 0.279
	K_M [µM]	379 ± 126	727 ± 178	1317 ± 451	1279 ± 899

	CP1 [µM]	0	1	3	6
mFSP1 C187S	v_{max} [mAU· s^{-1}·μM^{-1}]	2.20 ± 0.09	1.78 ± 0.07	1.69 ± 0.09	1.72 ± 0.34
	K_M [µM]	278 ± 31	309 ± 34	329 ± 46	1222 ± 381

	CP1 [µM]	0	5	30	60
hFSP1 WT	v_{max} [mAU· s^{-1}·μM^{-1}]	5.28 ± 0.14	4.37 ± 0.23	3.23 ± 0.34	2.94 ± 0.53
	K_M [µM]	203 ± 16	229 ± 36	354 ± 93	812 ± 273

	CP1 [µM]	0	1	3	6
hFSP1 S187C	v_{max} [mAU· s^{-1}·μM^{-1}]	4.99 ± 0.44	3.34 ± 0.28	2.56 ± 0.17	2.42 ± 0.22
	K_M [µM]	180 ± 52	117 ± 38	80.3 ± 24.9	129 ± 45

Shown are NADH consumption assays using recombinant, purified murine or human FSP1 (mFSP1, hFSP1) wildtype and the indicated point mutants. Resazurin was used as an electron acceptor. FSP1 oxidoreductase activities in the presence of CP1 were normalized to the respective enzyme activities measured in the presence of the solvent control DMSO (0.1% v/v). Data are means ± S.E. of n=3 biologically independent experiments (**A**) Increasing resazurin concentrations (12.5-300 µM), constant NADH (0.5 mM final); (**B**) increasing NADH concentrations (75-1200 µM), constant resazurin (0.2 mM final). Data are mean values ± S.E. of n=3 biologically independent experiments. Curves were fitted and parameters and standard errors were derived using the Michaelis-Menten model in GraphPad Prism software 7. K_M, Michaelis–Menten constant; v_{max}, maximum enzyme velocity. Solvent control samples

contained DMSO (0.01% v/v). mFSP1 and hFSP1 were used at final concentration of 3 µM or 0.3 µM, respectively.

SUPPLEMENTAL AUXILIARY FILES

Table S3: Source data.
